# Dense Collagen I as a Biomimetic Material to Track Matrix Remodelling in Renal Carcinomas

**DOI:** 10.1021/acsomega.4c04442

**Published:** 2024-09-28

**Authors:** Anuja Upadhyay, Deniz Bakkalci, Auxtine Micalet, Matt Butler, Marianne Bergin, Emad Moeendarbary, Marilena Loizidou, Umber Cheema

**Affiliations:** † UCL Centre for 3D Models of Health and Disease, Division of Surgery and Interventional Science, University College London, Charles Bell House, 43-45 Foley Street, W1W 7TS London, United Kingdom; ‡ UCB Pharma, 216 Bath Road, SL1 3WE Slough, United Kingdom; § Department of Mechanical Engineering, Roberts Building, University College London, WC1E 6BT London, United Kingdom; ∥ Division of Surgery and Interventional Science, University College London, Royal Free Campus, Rowland Hill Street, NW3 2PF London, United Kingdom

## Abstract

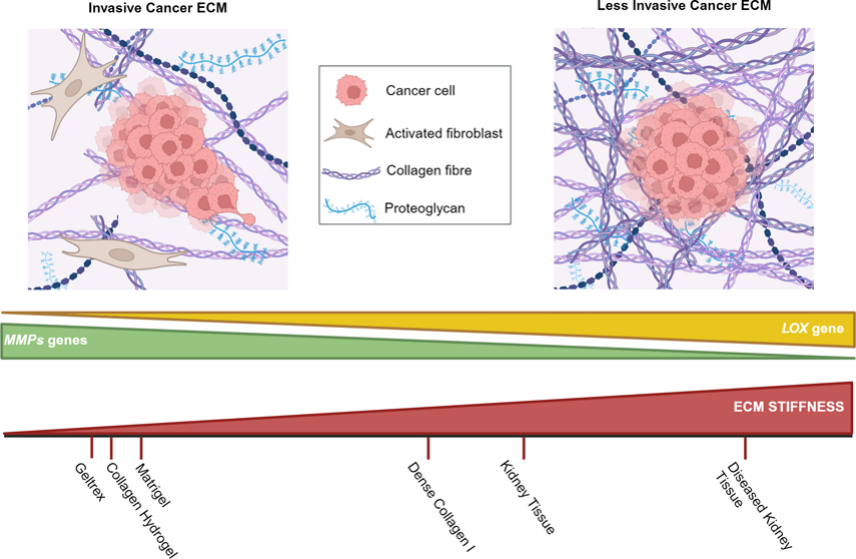

*Aims:* Renal tissue is a dynamic biophysical microenvironment, regulating healthy function and influencing tumor development. Matrix remodelling is an iterative process and aberrant tissue repair is prominent in kidney fibrosis and cancer. Biomimetic 3D models recapitulating the collagen composition and mechanical fidelity of native renal tissue were developed to investigate cell–matrix interactions in renal carcinomas. *Methods:* Collagen I and laminin hydrogels were engineered with renal cancer cells (ACHN and 786-O), which underwent plastic compression to generate dense matrices. Mechanical properties were determined using shear rheology and qPCR determined the gene expression of matrix markers. *Results:* The shear modulus and phase angle of acellular dense collagen I gels (474 Pa and 10.7) are similar to human kidney samples (1410 Pa and 10.5). After 21 days, 786-O cells softened the dense matrix (∼155 Pa), with collagen IV downregulation and upregulation of matrix metalloproteinases (MMP7 and MMP8). ACHN cells were found to be less invasive and stiffened the matrix to ∼1.25 kPa, with gene upregulation of collagen IV and the cross-linking enzyme LOX. *Conclusions:* Renal cancer cells remodel their biophysical environment, altering the material properties of tissue stroma in 3D models. These models can generate physiologically relevant stiffness to investigate the different matrix remodelling mechanisms utilized by cancer cells.

## 1 Introduction

**Figure 1:**
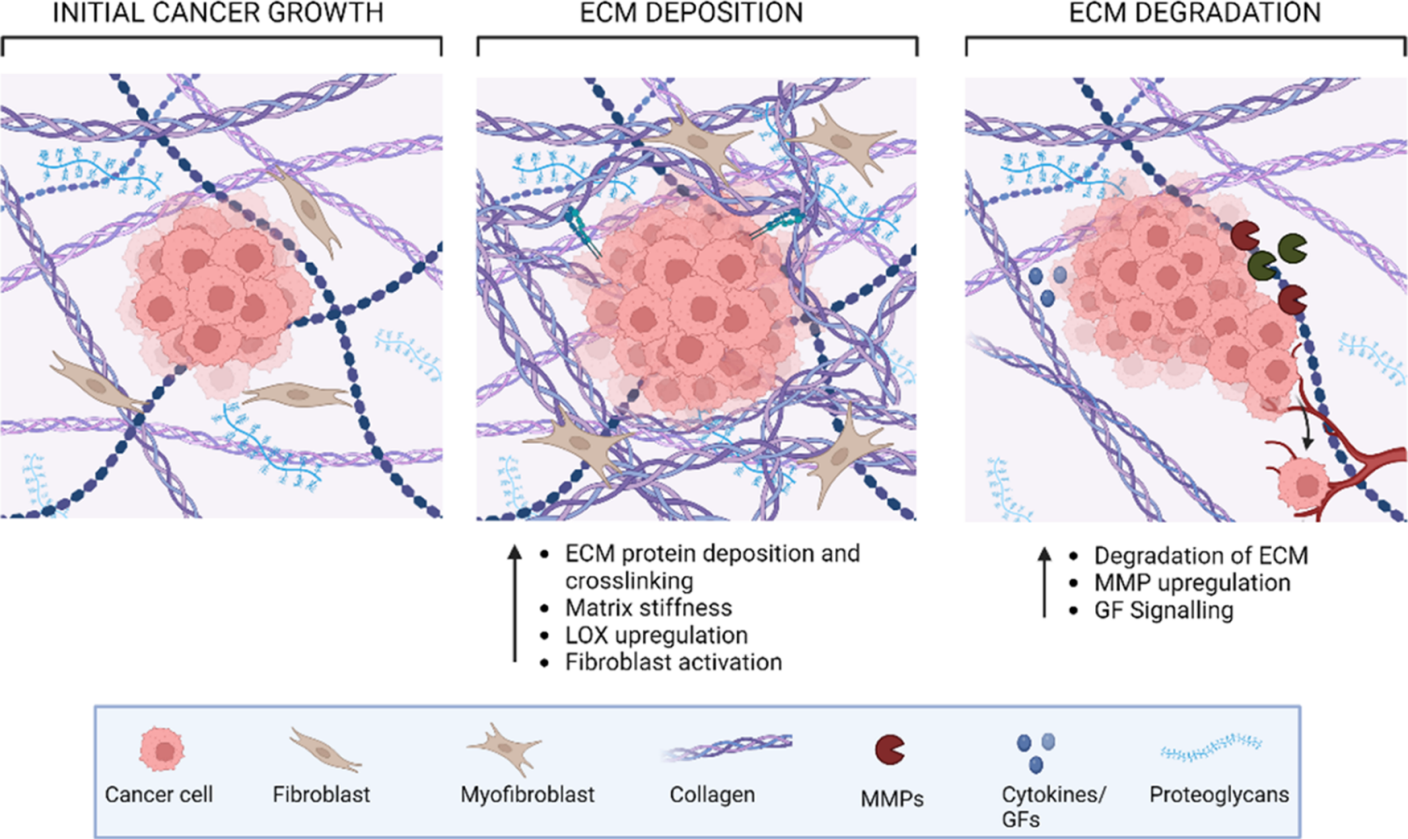
ECM remodelling in cancer. *In situ*, carcinoma and stromal cells are connected to the surrounding extracellular matrix. During cancer progression, activated tumor cells and myofibroblasts will remodel the surrounding ECM to promote malignancy. Tumour cells will release proteases that trigger proteolytic ECM degradation, inducing tumorigenic signaling which promotes cell proliferation and migration. It also involves increased secretion and deposition of ECM components, with key ECM enzymes such as LOX driving further cross-linking and alignment of collagen fibers. The reorganization of the ECM architecture and increased matrix stiffness will induce mechanosignaling by tumor cells. This will promote cancer invasion and support the tumorigenic environment through secretion of growth factors and cytokines promoting angiogenesis. Created using BioRender.com.

The extracellular matrix (ECM) is a dynamic and heterogeneous microenvironment, critical in maintaining tissue architecture and regulating key biochemical signaling cascades. It is composed of a complex array of fibrous proteins like collagen, basement membrane proteins and proteoglycans which can be collectively defined as the “matrisome”.^1,2^ In a healthy response to injury, the network regulates its protein deposition and degradation, supporting tissue repair and normal function. However, in a disease setting, aberrant matrix remodelling can disrupt organ function and impair drug delivery.^3^ In renal fibrosis and carcinomas, it is apparent that the tissue becomes stiff which also impacts the organ function. The key drivers of such remodelling are well documented and included dysregulated matrix deposition, cross-linking and random extracellular matrix orientation or remodelling (Figure 1). The deposition of extracellular matrix, namely collagen I as a majority constituent, is particularly relevant in cancer progression.^4^

Fibrosis, the thickening and scarring of tissue, is mainly characterized by the pathological accumulation of ECM proteins, epithelial-mesenchymal transition (EMT) of resident epithelial cells and inflammation.^5^ Key players include activated fibroblasts and immune cells which will interact with resident epithelial cells, releasing soluble factors i.e., TGF-β, fibroblast growth factor (FGF) and platelet derived growth factor (PDGF), to further stimulate myofibroblasts and drive the fibrotic phenotype.^6^ Kidney myofibroblasts often have diverse origins from tubular epithelial cells, endothelial cells and resident fibroblasts.^7^ The pathological principles underlying fibrosis are central in solid tumors, as desmoplasia and increased collagen cross-linking triggers cytoskeletal reorganization for the cells and mechanical stress in the stroma.^8^ Collagen cross-linking is driven by lysyl oxidase (LOX), transglutaminases (TGM) and advanced glycosylation end (AGE) products.^9^ In the fibrotic process, the TGF-β1/Smad3 or NF-κB signaling pathways can significantly upregulate LOX and TGM2 mRNA expression.^10,11^ The imbalance in matrix mechanics further promotes disease by inducing angiogenesis and hypoxia.^12,13^

Renal cell carcinomas (RCC) are one of the most common malignancies worldwide, with growing incidence and up to 17% of patients presenting with distant metastases at diagnosis.^14^ It often originates in the proximal tubular epithelium and is characterized by genetic abnormalities such as *VHL* or *MET* mutations and scar tissue formation.^15^ The asymptomatic nature in the early stages and weak response to radiotherapy and chemotherapy in advanced cases makes the prognosis poor as patients can often require aggressive treatment or transplantation.

There are five defined subtypes of RCC such as clear cell (ccRCC) which can account for up to 75% of cases and papillary (pRCC) which has a smaller share at 15%.^16^ The 786-O cell line from ccRCC is characterized by the chromosome 3p deletion and a mutation in the von Hippel-Lindau (VHL) tumor suppressor gene, leading to dysregulated hypoxia-inducible factors (HIFs).^17,18^ The HIF pathway activation leads to enhanced transcription of factors i.e., VEGF, Survivin, MMP2 and glycolytic enzymes. These are involved in survival, angiogenesis and metastasis in RCC. VHL also plays a key role in ECM remodelling, particularly fibronectin and collagen IV matrix assembly.^19^ Research suggests a HIF-independent role in binding to COL4A2 in the cytosol where it aids the assembly of collagen IV networks through mediated collagen hydroxylation.^20^

The ACHN cell line is derived from papillary renal cell carcinoma, however its characterization has been debated in literature. The cells are derived from pleural effusion and models metastatic disease, with research suggesting that it harbors a *c-met* polymorphism that is characteristic of papillary RCC.^21^ c-MET is a receptor tyrosine kinase which is key in cell differentiation and tissue repair in human tissue. Dysregulation has been implicated in tumor growth and invasion, as high c-Met expression correlates with pathological features and worse overall survival, as the HGF/c-MET pathway mediates Erk/MAPK, FAK and STAT3/5 activation.^22^

Malignant cells will tune their behavior to the 3D environment and concurrently drive disease by altering the architecture.^3,23^ The role of cancer associated fibroblasts (CAFs) in ECM deposition, altering expression of matrix metalloproteinases (MMPs) and chemokines to support tumor development is well established.^24,25^ However, cancer cells themselves also influence the remodelling of both the interstitial matrix and basement membrane, in order to promote invasion and metastasis.^26^ These network and cell-based changes have been studied in the past, looking at alterations in stiffness involving cell generated forces and shifts in ECM mechanics. The mechanical properties of the ECM such as collagen fiber stiffness can influence tumor progression and promote an aggressive phenotype through mechanotransduction pathways. This can involve integrin signaling, YAP/TAZ activation, cytoskeletal reorganization.^27^ Integrins are cell surface receptors that mediate cell-ECM adhesion and increased stiffness will enhance receptor activation. Consequently, this will activate focal adhesion kinase (FAK) and downstream signaling pathways such as the MAPK/ERK and PI3K/AKT pathways, which promotes cell survival and migration.^27^ ECM stiffness can also induce EMT which is controlled by transcription factors such as Snail, Slug, and Twist, and these are regulated by mechanical cues from the ECM.^28^

**Figure 2:**
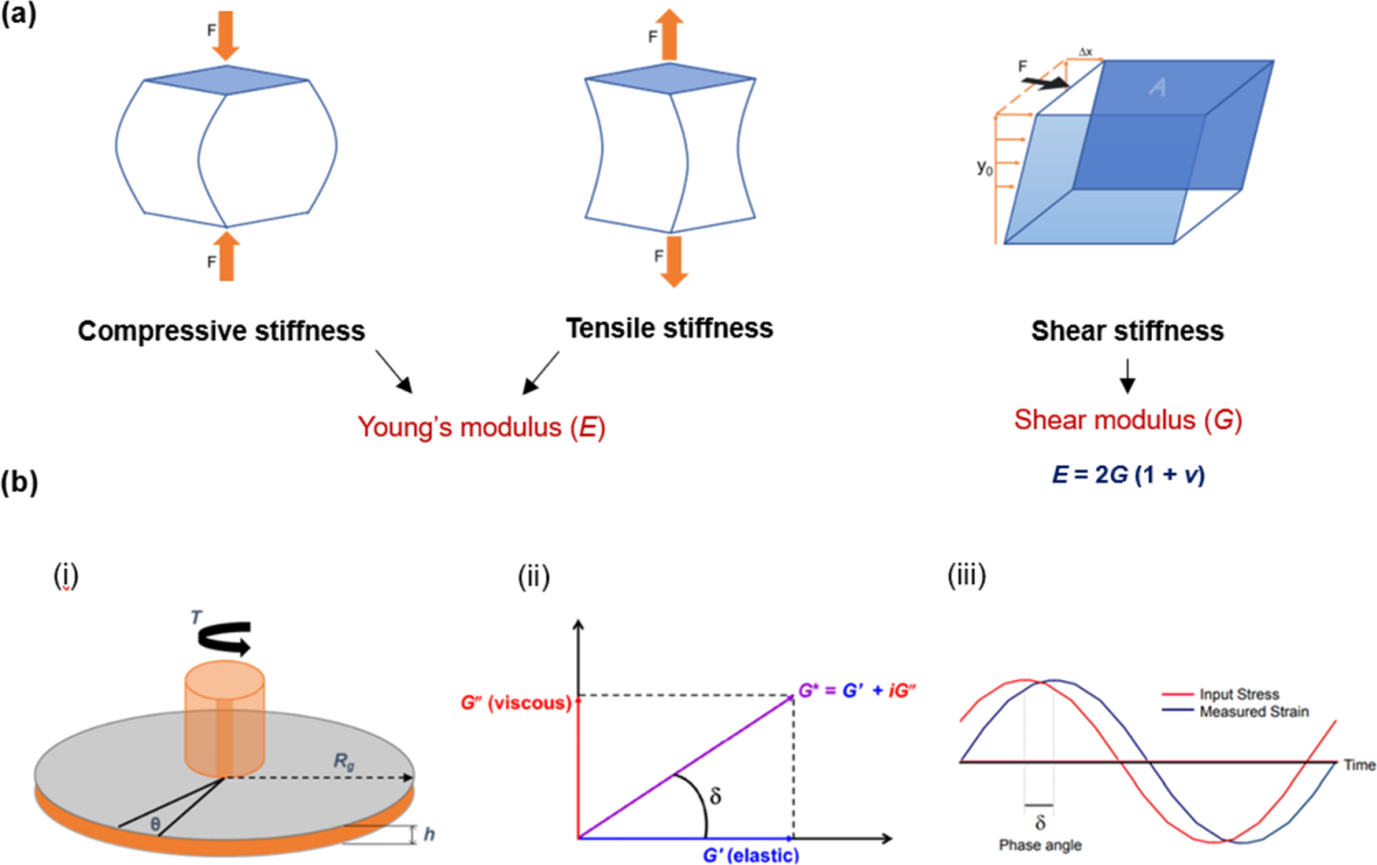
Methodologies for measuring mechanical properties. (a) The mechanical properties of solid materials can be measured using different moduli. Compressive or tensile testing, where force is applied lengthwise, will determine the Young’s modulus (*E*). Shear testing will determine the shear modulus (*G*), where force is applied parallel to one of its surfaces. In isotropic materials, the shear modulus (*G*) and Young’s modulus (*E*) are connected using the equation *E* = 2*G* (1 + (*v*)), (*v*) defines the Poisson’s ratio which is the deformation (expansion or contraction) of a material in directions perpendicular to the direction of loading. (b) (i) Representation of the shear rheometer geometry, the sample sits between parallel plates and variables such as gap height (h) and torque (*T*) can be controlled. (b) (ii) Mechanical properties determined by shear rheology is the shear modulus (G*) which is calculated by the viscous (*G*″) and elastic (*G*′) modulus, and their relationship is defined by the phase angle (δ). (b) (iii) Graphical representation of the phase angle, an arbitrary unit calculated by the lag between the input stress and measured strain from rheological testing. Figure graphics created using Microsoft Powerpoint.

Stiffness is defined as a material’s ability to resist deformation. This is measured by various elastic moduli: compression, shear, and tension (Figure 2a) which is known as Young’s modulus or Shear modulus.^29^

Due to the different modalities used to measure elasticity such as atomic force microscopy and rheology, there is a lack of consensus regarding the stiffness of tissues and biomaterials. It has been established using ultrasound-based techniques and *ex vivo* testing that kidney tissue stiffness measures around 4 kPa and increases with fibrosis up to 10 kPa.^30−32^ In biomaterials, the literature values vary. Mechanical properties of collagen and natural protein-based models range from 30–400 Pa depending on the fabrication and testing parameters.^33−37^ Furthermore, the tensile modulus of collagen I networks has been shown to be much larger than the compression modulus, under the same experimental parameters.^38^ This may be because of the changes occurring in the network during compression, causing disorientation and rearrangement of the fibers. The methods measure different forms of elasticity and can be conducted on post-mortem tissue or in living patients. Therefore, the data is scattered, and the output is greatly influenced by factors such as patient age or gender or orientation of ultrasound probes. It is fundamental to standardize the measurement techniques for tissue stiffness to advance our knowledge and generate reliable results so that we can utilize this data for clinical practice. Establishing reference values for tissue stiffness would enhance the accuracy of bioengineering research and could even help in assessing tumor progression or response to treatment.

Elasticity is the ability for a material to return to its original shape after the stress is released. This describes the relationship between stress and strain. Stress is the ratio of force over the area its applied to and strain is a unitless measure of deformation representing displacement relative to the original shape. Nevertheless, this does not fully describe the physical properties of the material as it does not consider the nonlinear nature of their behavior. Soft tissues, ECM and engineered tissue constructs should be defined as viscoelastic. Viscoelasticity is a property of materials that possess both elastic and viscous energy potential and the relationship between the two determines the type of material it is.^39^

The cells and matrix proteins work together as a nonlinear material where its inherent elastic and viscous properties can change with time and the stress applied. This is unlike linear elasticity, which describes a linear regression in the relationship between stress and strain.^40^

Among the most common methods for evaluating the mechanical properties of engineered constructs is oscillatory shear rheology (OSR) which applies a sinusoidal shear stress profile to a sample between two plates (Figure 2bi) and measures its complex shear rigidity (G*) which can be separated into elastic storage (*G*′) and viscous loss (*G*″) moduli. The viscous energy is the lost dissipated energy in the material and the elastic potential refers to the stored energy that the material possesses.
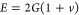

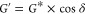

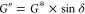

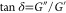
These equations describe the relationship between the elastic and viscous moduli in the shear complex modulus (Figure 2bii). A sample behaves as a fluid if *G*″ > *G*′ or tan δ > 1, and it shows gel-like behavior with stability if *G*′ > *G*″ or tan δ < 1, respectively. Phase angle (δ) is the lag between input stress and measured strain (Figure 2biii). The phase angle ranges from 0 to 90°, informing us if the material deforms elastically like a solid or flows like a viscous material. Semisolids and gels are known to lie between 0 and 45°. In practice, *G*′ values in the LVE region represent the stiffness of the sample or the gel strength. This method enables more in-depth material characterization and is utilized in this study to determine the mechanical properties of bioengineered constructs and tissues.

ECM composition and stiffness are very important in normal homeostasis and disease, in particular kidney cancer and fibrosis. We aim to study and track the mechanical and biochemical changes in the remodelling of a bioengineered collagen I matrix over 21 days, driven by renal carcinoma cells. Comparing renal cancer phenotypes using 786-O cells, derived from invasive clear cell renal carcinoma (ccRCC) to ACHN cells which are established from pleural effusion of papillary RCC.^41,42^

Bioengineered models provide a more accurate representation of the physiological and topographical features of the human ECM. It also allows researchers to precisely control the microenvironment which is crucial for understanding how cells respond to different physical and biochemical cues. The kidney is a highly complex system and incredible advancements have been made in developing *in vitro* models such as animal models, organoids, microfluidic devices, bioprinting and hydrogel-based models. However, they often must focus on one aspect of the disease to develop a system that recapitulates a part of the tumor microenvironment such as the architecture or interactions between specific cell populations. Organoids present great potential as a tool for therapeutic testing i.e., for drug sensitivity and in mimicking nutrient gradients in tumors, however they are extremely labor and time intensive.^43^ Microfluidic devices have been used to study kidney function and nephrotoxicity however they are limited by technical expertise and availability.

Collagen-based models can provide a more natural protein ECM platform and many groups aim to tune the material stiffness and fabrication of these models using chemical cross-linking.^44,45^ However, this may result in a toxic environment for the cells and therefore the cells are often seeded on top of the surface. Our model embeds the cells within the matrix, more similar to how the cells would live in the body’s ECM and this allows for us to study more biologically accurate behaviors by the cells. Our work also presents a method that can allow for us to measure and determine the mechanical properties of bioengineered tissue models and compare directly to human tissue from the diseases. It is important to note that several methods for fabricating collagen-based models exist such as gel-aspiration and ejection,^46^ and we have utilized the RAFT system. This system ensures robust cell viability and allows for further downstream analysis such as imaging, molecular expression and biomechanical measurements over time. The behavior of cancer cells in this study will be explored using a well characterized and established 3D *in vitro* model, previously used to investigate various cancers such as bone, colorectal and pancreatic malignancies.^24,47−49^

## 2 Materials and Methods

### 2.1 Cell Culture

Two human renal cell carcinoma cell lines were used; 786-O and ACHN (European Collection of Authenticated Cell Cultures, Public Health England, UK). All cancer cell lines were cultured in RPMI-1640 medium 1× with l-Glutamine (Gibco through Thermofisher Scientific, Loughborough, UK) supplemented with 10% volume Foetal Bovine Serum (FBS), 100 units/mL penicillin and 100 μg/mL streptomycin (Gibco through Thermofisher Scientific, Loughborough, UK). Cells were cultured at 5% carbon dioxide (CO_2_), 95% atmospheric air and at 37 °C and passaged regularly in 2D monolayers. 3D set ups were conducted under passaged number 20 for all cultures.

### 2.2 3D Model Fabrication

**Figure 3:**
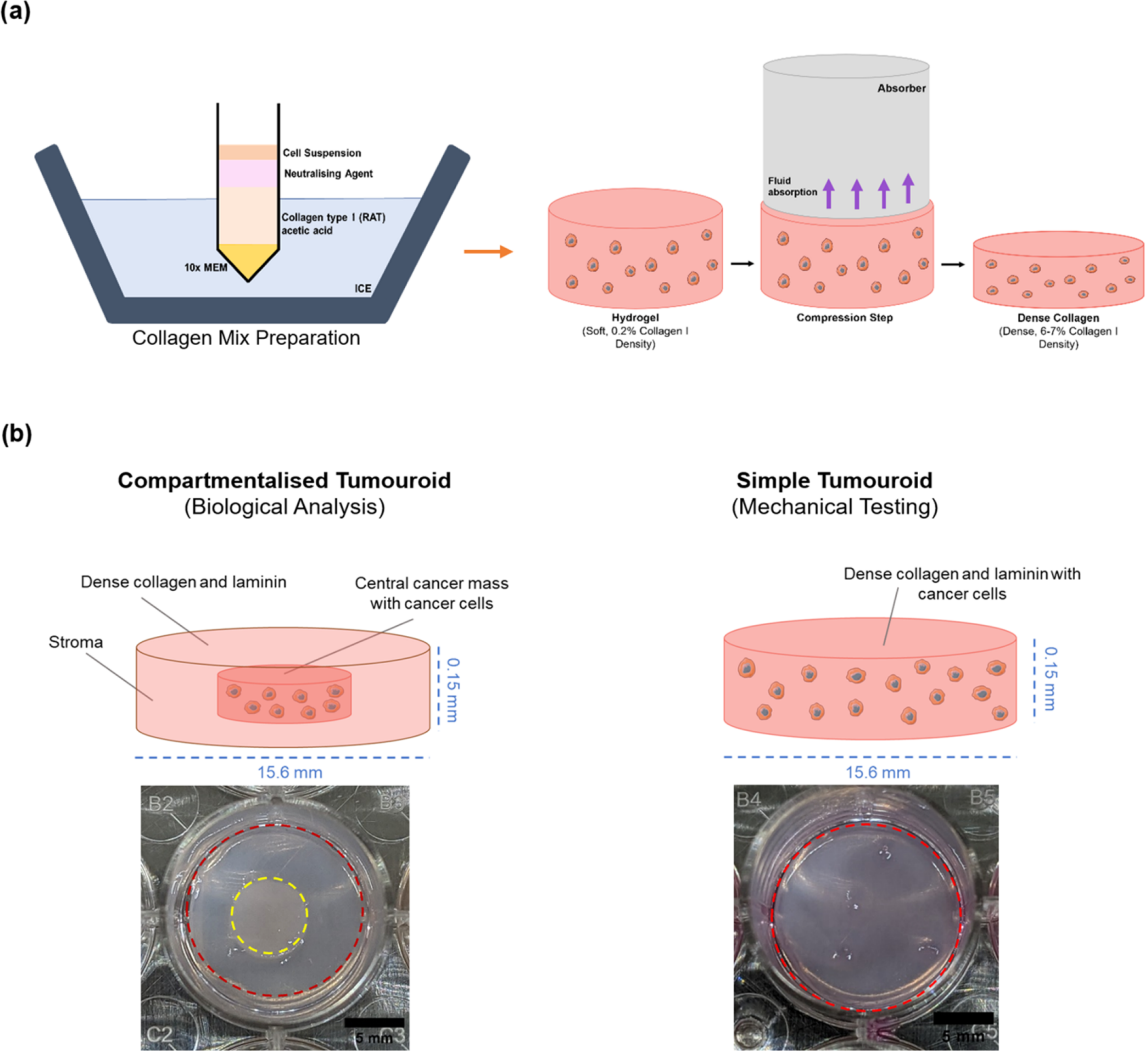
Key experimental methodologies. (a) Fabrication of type I collagen hydrogels, followed by compression of gels using absorbers. (b) Bulk stiffness measurements and gene analysis were conducted on dense collagen gels containing cancer cells (referred to as simple “tumouroids”). Invasion quantification was performed on complex “tumouroids.” The compartmentalized tumoroid models consisted of compressed collagen with cancer cells in a central cancer mass surrounded by an acellular stroma. Photograph courtesy of Anuja Upadhyay, Copyright 2024. Figures created using Microsoft Powerpoint.

All 3D simple and compartmentalized tumoroids were fabricated with monomeric type I collagen (2 mg/mL) (First Link, Birmingham, UK) using the RAFT protocol (Lonza, Basel, Switzerland) which provides required volumes for all reagents (Figure 3). This begins with mixing 10x Minimal Essential Medium (MEM) (Gibco through Thermofisher Scientific, Loughborough, UK) with collagen I and neutralizing solution (N.S.). N.S. was made from 16.5% v/v 10 M NaOH (Sigma-Aldrich through Merck, Gillingham, UK) and 10 M HEPES Buffer (Gibco through Thermofisher Scientific, Loughborough, UK). Laminin (Corning through Thermofisher Scientific, Loughborough, UK), a basement membrane protein is added at a 50 μg/mL concentration. All reagents were kept on ice until cells were added, always working in sterile conditions (Figure 3).

Simple tumoroids were set up at a volume of 1.3 mL, with a seeding density of 2.5 × 10^5^ cells, in 24-well plates (Corning through Thermofisher Scientific, Loughborough, UK), polymerized in a 37 °C incubator for 15 min to create a collagen I hydrogel. Excess fluid is removed by plastic compression using RAFT absorbers (Lonza, Basel, Switzerland) to create a dense collagen model, increasing the collagen density from 0.2 to 4%.

The complex tumoroid design describes a stromal compartment with a tumor mass embedded within. The tumor mass is created by adding collagen/cancer cells gel mix into a 96-well plate and polymerizing for 15 min at 37 °C. This is followed by plastic compression by absorbers.

The first stromal layer is 650 μL added to the bottom of a 24-well plate (Corning through Thermofisher Scientific, Loughborough, UK) and polymerized in the incubator for 15 min at 37 °C. Then, the tumor mass was carefully cast on top of the set gel, followed by 650 μL of the stromal mix to embed the mass. Following polymerization for 15 min at 37 °C, plastic compression was conducted for 15 min. All tumoroids were hydrated with 1 mL media, 50% of media was changed every 48 h. All tumoroids were cultured at 37 °C and 95% atmospheric air. All cellular models are cultured for 21 days in their respective media with ascorbic acid supplement (50 ng/mL) (Merck Life Sciences, Dorset, UK) to support collagen deposition.

### 2.3 Rheology

Mechanical testing experiments were all performed on a Kinexus Pro+ rheometer (Netzsch, Selb, Germany), which executes stress-controlled, shear rate- and strain- controlled oscillations to test material properties. All tests were conducted using a roughened immersion well geometry and a 20 mm roughened steel plate top geometry.

The rheometer has a torque range of 1.0 nNm to 225 N m. Gels were tested at a 0.2 mm gap to ensure a no-slip boundary between the samples and geometry. To prevent drying of the sample during all testing, 500 μL of media was used/maintained after each test and a solvent trap was used to cover the setup. Human cancer tissue was cut into 1.5 cm (length and width) pieces and tested at a gap size of 2 mm. Main measures for biophysical characterization are the shear elastic modulus, LVER, phase angle and the shape of curve. Data was analyzed with the software supplied by the instrument manufacturer (rSpace for Kinexus, Netzsch).

#### 2.3.1 Strain/Amplitude Sweep

An oscillatory strain sweep was conducted, in which the shear stress varied over a range of 0.1–100.0% and the amplitude refers to the maximum of the oscillatory motion. This testing was performed at 37 °C with a constant frequency of 1.0 Hz. Experiments were repeated three times to ensure reproducibility of results. Ten samples per decade were measured for each test and normal force was kept under 1 N.

The linear region was determined by the drop in elastic modulus plateau region over the increasing strain rate and further validated using the rSpace software, which calculated the LVER. Flow points were estimated by the crossover point between the *G*′ and *G*″ curves. Average stiffness was calculated by averaging the *G*′ values in the linear region for an average value per sample, with 3 testing runs conducted per each sample.

### 2.4 RNA Extraction and Quantitative Polymerase Chain Reaction (qPCR)

RNA was extracted from 3D collagen I constructs with 786-O and ACHN cells. Phase separation TRI Reagent and chloroform method was used.^50^ cDNA was reverse transcribed using the High-Capacity cDNA Reverse Transcription Kit (Applied Biosystems through, Fisher Scientific, Loughborough, UK). Primers used for qPCR can be found listed in Table S1. qPCR was performed using iTaq Universal SYBR Green Supermix (Applied Biosystems through, Fisher Scientific, Loughborough, UK). Three technical replicates were performed. Relative gene expression was calculated using the ΔCt method, normalizing to housekeeping gene *GAPDH*.

### 2.5 Immunofluorescent Staining and Imaging

3D cultures were fixed using 10% w/v neutrally buffered formalin (Genta Medical, York, UK) for 30 min and then washed and stored in PBS (Gibco, through Fisher Scientific, Loughborough, UK). Before staining, all samples were blocked for 1h using 0.2% triton X-100 and 1% bovine serum albumin (BSA) (Sigma-Aldrich, Dorset, UK) in PBS. Then, the primary antibody, diluted in blocking solution was applied to the samples. They were incubated overnight at 4 °C. The secondary antibody incubation was carried out the next day for 2.5 h, at room temperature. All antibodies and dilutions are provided in Table S2. A DAPI counterstain was also applied 20 min before imaging (NucBlue, Invitrogen through Fisher Scientific, Loughborough, UK). Samples were imaged on the Zeiss AxioObserver and Zeiss ZEN software (Zeiss, Oberkochen, Germany). Image analysis was conducted using ImageJ software.

### 2.6 Statistical Analysis

All data was analyzed and visualized using GraphPad Prism 10 software. All *n* numbers, *p*-values and tests conducted are mentioned in the respective figure captions.

## 3 Results

### 3.1 Material Properties of Bioengineered Models and Tissue

**Figure 4:**
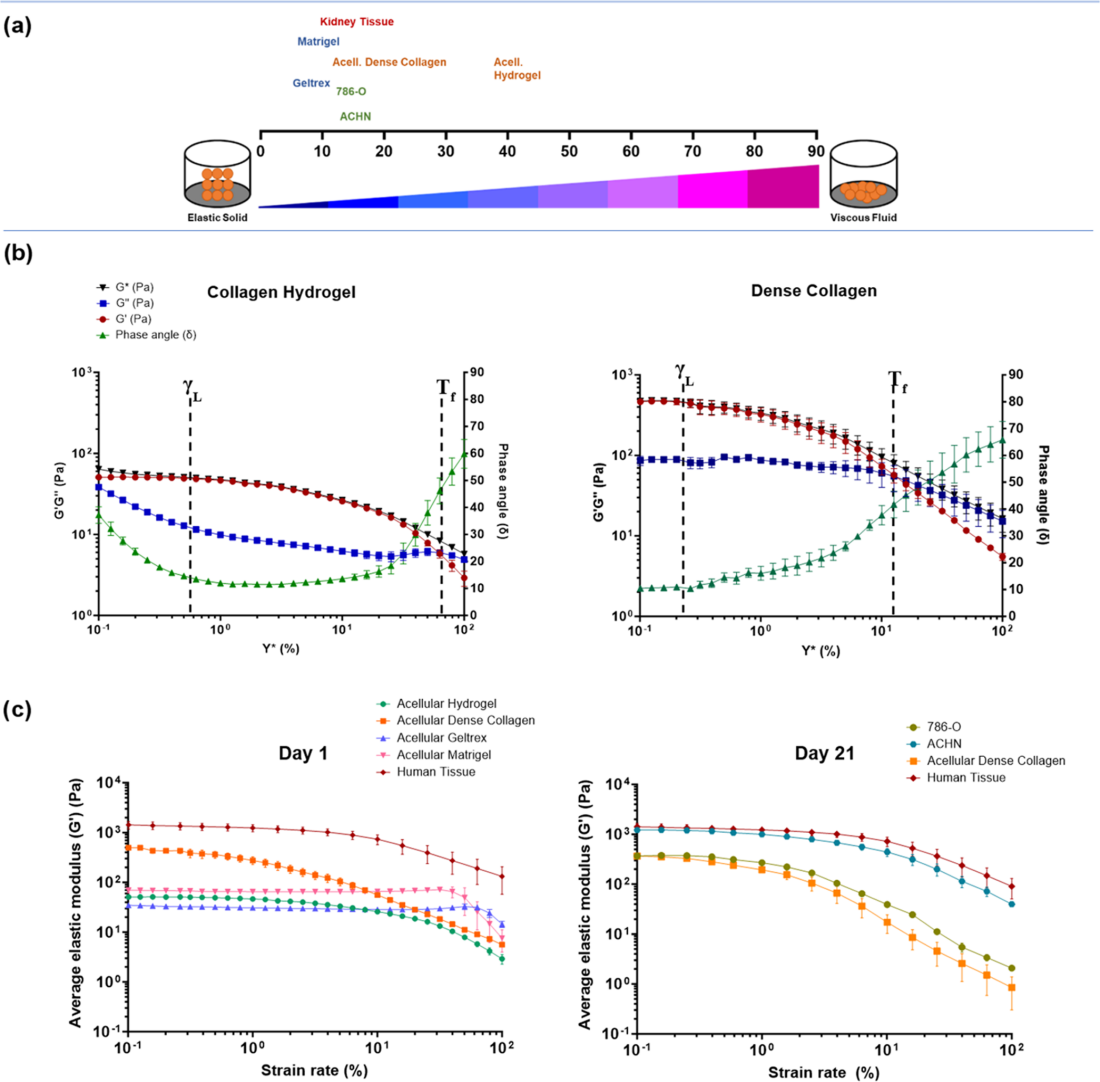
Material properties of bioengineered models and human tissue. (a) Phase angle (δ) chart of different biomaterials and tissues, measured on day 1. (b) Amplitude sweep testing for acellular collagen I hydrogels and dense gels on day 1; with phase angle (δ), shear complex modulus (G*), shear elastic modulus (*G*′) and the viscous modulus (*G*″). The end of the linear viscoelasticity region (γ_L_) and the flow points (*T*_f_) for the materials are annotated. (c) Comparison of linear viscoelasticity regions for different biomaterials at day 1 and day 21, mapping the shear elastic modulus (*G*′) values over increasing strain rates, *n* = 3 for all samples.

The mechanical properties of the dense collagen I model were measured using rheology and directly compared to commonly used bioengineered scaffolds and human kidney tumor tissue. Acellular collagen I hydrogels are significantly softer, with an average phase angle of 37.2 and an average elastic modulus of 51.08 ± 5.16 Pa, indicating greater viscous potential. Whereas, acellular dense collagen I is much stiffer, with an average shear elastic modulus of 474.03 ± 42.5 Pa and an average phase angle of 10.67. The lower phase angle indicates the material behaves more like an elastic solid and its mechanical profile is closer to the measured human renal tissue. Human kidney tissue measures much stiffer, averaging at 1.4 kPa however its phase angle is much closer to the dense collagen I gels at 10.5. This suggests that the dense collagen model is able to more closely mimic the shear elastic properties of human tissue in comparison to other tested biomaterials. It would be beneficial to test more human tissue to account for the variability seen within the organ structures. The linear viscoelasticity region (γ_L_) of dense collagen gels (Figure 4b) is much shorter, indicating that less shear strain is required for the material to begin to fail as it is no longer able to withstand the deformation.

Geltrex and Matrigel are commonly used 3D biomaterials, with a similar shear stiffness to acellular collagen I hydrogels, at 32.9 ± 0.72 and 72.1 ± 8.61 Pa respectively. However, the determined phase angle is much lower (Figure 4a), which indicates that it behaves more like a solid in comparison to the hydrogels with a greater water content.

On day 1, the shape of the linear viscoelasticity curve of human kidney tumor samples is comparable only to the acellular dense collagen model, unlike other biomaterials (Figure 4c). However, after 21 days of culture, the introduction of renal cancer cells (ACHN or 786-O) results in significant changes in the viscoelastic behavior due to cell remodelling of the collagen. In particular, the ACHN cells remodel the matrix and stiffen the material, to mimic the viscoelastic nature of human tissue more closely. However, 786-O cells remodel the matrix and soften it to behave similarly to the acellular collagen dense models. Overall, across the different cancer cell types and human tissue there is significant variability in the rheological properties but our method shows strong reproducibility within the populations. This highlights how the choice of biomaterial is critical when investigating the behavior of cancer cells in 3D.

### 3.2 786-O Renal Cancer Cells Soften the Matrix over Time

**Figure 5:**
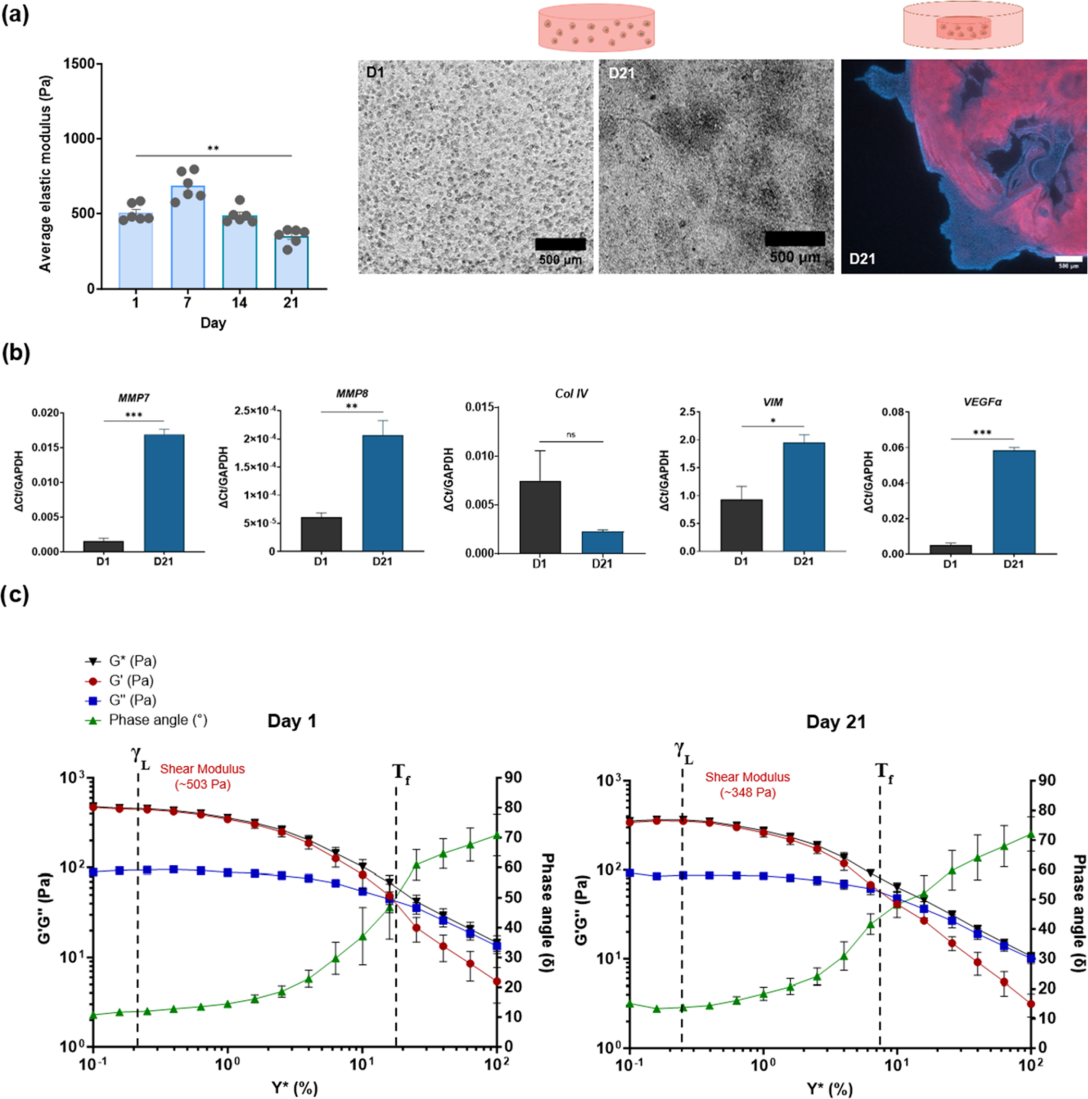
Behavior of 786-O cells in dense collagen I matrix over time. (a) Average stiffness of 786-O simple tumoroids, One-way ANOVA statistical testing conducted. Phase contrast images of simple tumoroids from day 1 to day 21, scale bar (500 μm) and fluorescence imaging of cancer mass and stroma boundary at day 21 using DAPI and Cytokeratin-8, scale bar (500 μm). (b) Changes in gene expression from Day 1 to Day 21, normalized to housekeeping gene *GAPDH*. *n* = 6 for all conditions. Shapiro- Wilks test and One-Way ANOVA and Tukey’s multiple comparisons or *t* tests, respectively. (a) p-values **p < 0.004 (b) *p < 0.02, **p < 0.005, ***p < 0.0002. (c) Amplitude sweep testing for 786-O collagen I dense gels on day 1 and day 21; with phase angle (δ), shear complex modulus (G*), shear elastic modulus (*G*′) and the viscous modulus (*G*″). The end of the linear viscoelasticity region (γ_L_) and the flow points (*T*_f_) for the materials are annotated. (*n* = 3 for both time points).

Over time, 786-O cells softened the collagen I matrix, from 503.9 ± 52.5 to 348.3 ± 47.2 Pa. The matrix degradation by invasive clear cell renal carcinoma cells is supported by significant upregulation of genes, including *MMP7* and *MMP8* (Figure 5b), and downregulation of the key basement membrane protein collagen IV (*COL4*) gene. Molecular expression of vimentin (*VIM*) and *VEGF*α increases as the 786-O cells proliferate and degrade the matrix over time.

The linear viscoelasticity region (γ_L_) is greater in cultures at day 21 (Figure 5c), indicating that the material has become softer. Due to the increase in the viscous modulus, it can internally stabilize its architecture and requires a greater strain rate for the material to begin losing its structural integrity. While the structural strength after the LVE region starts to diminish, it still predominantly displays solid matter properties. The flow point (*T*_f_) occurs at a much lower strain rate at day 21, which suggests that less strain is needed for the viscous energy to dominate and for the material to begin to flow.

The cell driven changes in biomechanical properties are further demonstrated by the change in phase angle toward a more viscous potential, from day 1 at 10.77 ± 0.48 to 15.14 ± 1.38 at day 21.

Moreover, as the 786-O cells invade into the acellular stroma by D21 from the boundary of the central cancer mass, we can visually observe the loss of cytokeratin-8 positive staining. This can indicate a change in expression of cytokeratin as the cancer cells begin to invade and undergo EMT. In addition, the matrix degradation driven by 786-O cells is evident by the presence of empty spaces within the cancer mass by day 21.

### 3.3 ACHN Renal Cancer Cells Stiffen the Matrix over Time

**Figure 6:**
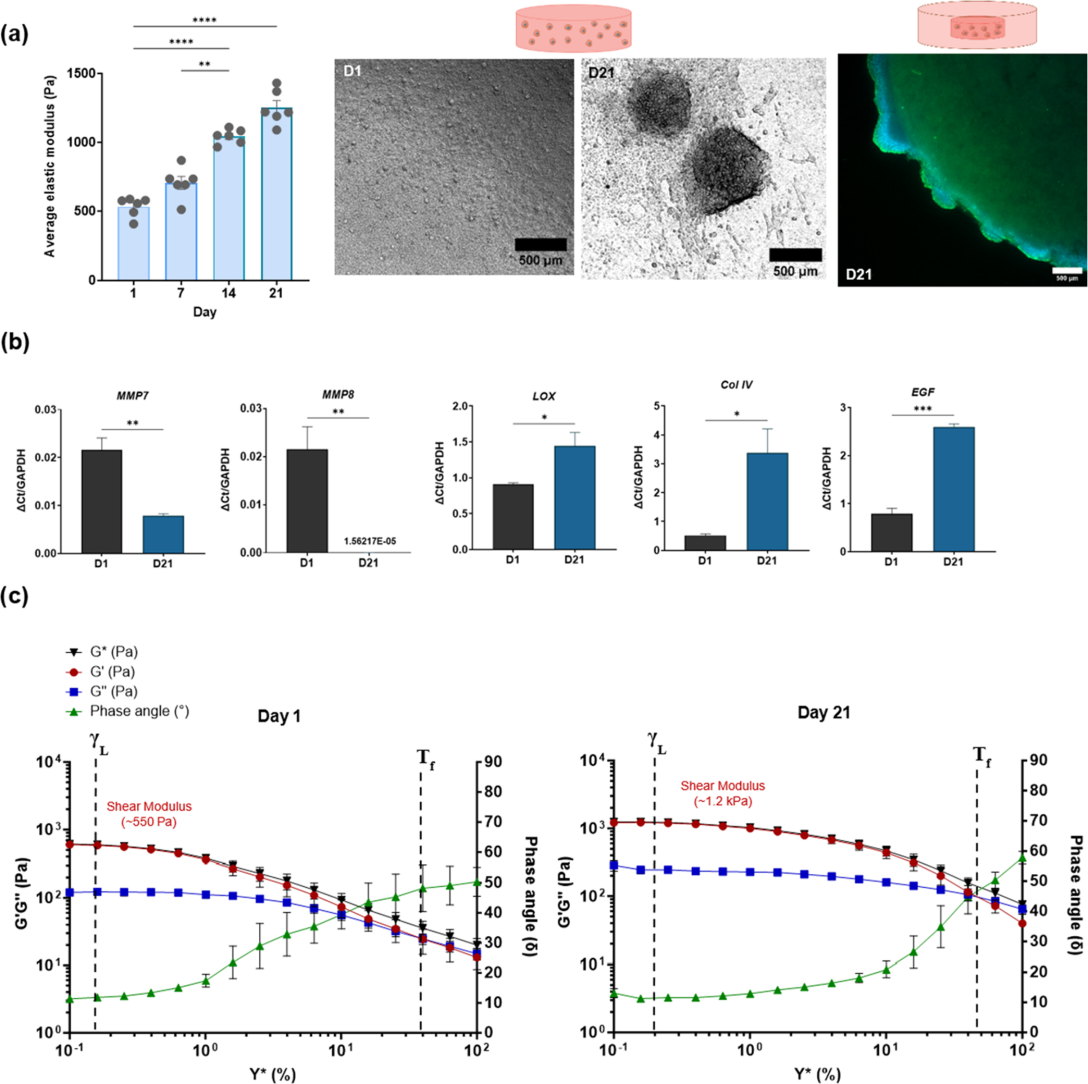
Behavior of ACHN cells in dense collagen I matrix over time. (a) Average stiffness of ACHN simple tumoroids, One-way ANOVA and Tukey’s multiple comparisons testing conducted. Phase contrast images of simple tumoroids from day 1 to day 21, scale bar (500 μm) and fluorescence imaging of cancer mass and stroma boundary at day 21 using DAPI and Cytokeratin-8, scale bar (500 μm). (b) Changes in gene expression from Day 1 to Day 21, normalized to housekeeping gene *GAPDH*. *n* = 6 for all conditions. Shapiro- Wilks test and One-Way ANOVA and Tukey’s multiple comparisons or *t* tests, respectively. (a) *p*-values **p < 0.0019, ****p < 0.0001. (b) *p < 0.05, **p < 0.006, ***p < 0.0002. (c) Amplitude sweep testing for ACHN collagen I dense gels on day 1 and day 21; with phase angle (δ), shear complex modulus (G*), shear elastic modulus (*G*′) and the viscous modulus (*G*″). The end of the linear viscoelasticity region (γ_L_) and the flow points (*T*_f_) for the materials are annotated. (*n* = 3 for both time points).

ACHN renal cancer cells stiffen the matrix over 21 days (Figure 6a), from 532.9 ± 64.34 to 1253.2 ± 114.2 Pa measured by shear rheology, forming spheroids in the dense collagen matrix that reach over 400 μm in diameter. These cancer cells, derived from a metastatic clear cell renal carcinoma, remodel the biomimetic matrix, with significant gene upregulation of collagen IV (*COL IV*) and *LOX*, a key enzyme driving cross-linking of collagen proteins. The role of MMPs in matrix remodelling, specifically −7 and −8, is further supported by the action of ACHN cells. With an increase in material stiffness, MMP expression is downregulated (Figure 6b). Concurrently, the matrix stiffening by ACHN cells is promoted by gene upregulation of *EGF*, a key pro-fibrotic growth factor in the tumor microenvironment.^26^

The end of the linear viscoelasticity region and the flow point of the ACHN cultures at day 21 requires a greater strain rate in comparison to day 1, which may suggest a softer structure. However, the significant increase in the elastic modulus demonstrates the nuanced remodelling of proteins and 3D architecture which is driven by different cancer cells. Over time, the phase angle of the matrix shifts closer toward an elastic solid potential, from 12.33 ± 0.55 to 10.93 ± 0.77. This suggests that the ACHN cells are remodelling the collagen matrix into a more solid material with less viscous energy.

### 3.4 Matrix Remodelling is Temporal in Nature and Linked to Invasive Capability

**Figure 7:**
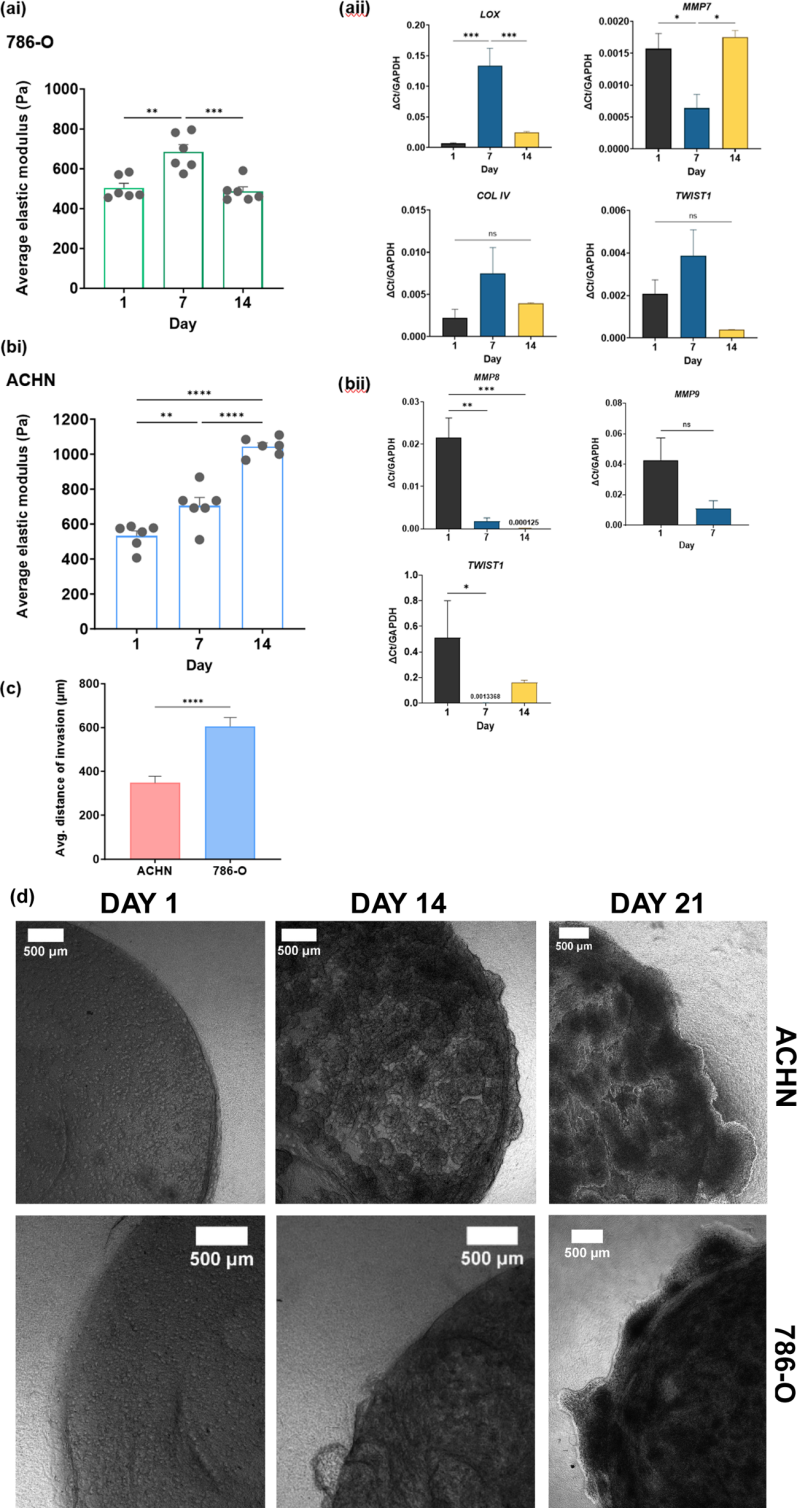
Temporal remodelling of the dense collagen I matrix by renal cancer cells. (ai) Average stiffness of 786-O simple tumoroids over 14 days. (aii) Changes in gene expression over the time points, values normalized to housekeeping gene *GAPDH*. Shapiro- Wilks test and One-Way ANOVA and Tukey’s multiple comparisons or *t* tests, respectively. (ai) *p*-values **p < 0.002, ***p < 0.0005 (aii) *p < 0.03, ***p < 0.001. (bi) Average stiffness of ACHN simple tumoroids over 14 days and (bii) Changes in gene expression over the time points, values normalized to housekeeping gene. Shapiro- Wilks test and One-Way ANOVA and Tukey’s multiple comparisons or *t* tests, respectively. (bi) *p*-values **p < 0.007, ****p < 0.0001 **(bii)** *p < 0.02, **p < 0.001, ***p < 0.0001. (c) Average distance of invasion from tumor-stroma boundary of day 21 tumoroids, *n* = 3 per cell line. *t* test analysis, p-value ****p < 0.0001. (d) Representative phase contrast images on 786-O and ACHN compartmentalized tumoroids at day 1, 14, and 21. Scale bar (500 μm).

Cell behavior in a 3D environment is constantly evolving, and matrix remodelling is a cyclical process. Certain cancer cells will undergo phases of stiffening the matrix as they proliferate and then begin degrading the collagen architecture to create space for them to grow and invade. 786-O cells demonstrate this cyclical nature (Figure 7ai), as the matrix stiffness increases to 684.1 Pa on average at day 7 and then decreases to approximately 486.8 Pa by day 14.

This shift in biomechanical properties can be tracked by fluctuations in gene expression with significant upregulation in *LOX* and downregulation of *MMP-7* at day 7. In addition, trends in molecular expression of other key markers such as collagen IV (*COL IV*) and *TWIST1* can be seen in the 786-O dense collagen gels. For example, there is an increase at day 7 and subsequent drop by day 14, which is reflected in the mechanical properties of the model. *TWIST1* gene encodes Twist related protein-1, a key transcription factor implicated in tumor progression via EMT and ECM stiffness^51,52^ and our findings suggest that its expression is more specific to the cancer cell phenotype.

The invasive nature of renal cancer cells is also highlighted through the model, with 786-O cells invading into the acellular stroma at a greater distance of 591.4 μm on average compared to ACHN cells at 369.2 μm (Figure 7d,e). The 786-O cells, more invasive in nature, will degrade the matrix during proliferation of cancer cells, aided by the upregulation of MMPs.

## 4 Discussion

Cancer cells alter the ECM, in order to grow through the tissue and spread the malignancy. The plasticity of the matrix is critical for renal carcinoma progression, and this is evident through the behavior of different tumor cells (786-O and ACHN) through the dense collagen I matrix. This model allows us to establish the differences in how cancer cells interact with the 3D environment and remodel the matrix as they proliferate. It highlights how different cancer cells will use varying mechanisms, for example more invasive cancer cells promote ECM degradation, in comparison to ACHN that model more papillary carcinoma and will promote desmoplasia.

Using shear rheology, the mechanical profiles of different bioengineered materials and human tumor samples (Table 1 and Supporting Figure 1) can be directly compared. Previous research investigating the mechanics of renal tissues measured tissue stiffness ranging from 0.5–15 kPa, as stiffness increased with severity of fibrosis.^53−56^ Bioengineered materials, mainly hydrogels, have also been explored in the literature and sit around 10–258 Pa.^35−37,57^ However, due to differences in material composition, mechanical testing methodologies and their parameters, the measured values vary. The previous findings have established a baseline for our work as we have noticed similar patterns in the material properties of our samples using shear rheology.

**Table 1:** Mechanical Properties of Bioengineered Models and Human Tissue Measured Using Shear Rheology

**material**	**shear elastic modulus (***G***′) ± s.d.**	**phase angle (δ) ± s.d.**
matrigel	72.1 Pa ± 8.61	5.02 ± 0.35
geltrex	32.9 Pa ± 0.72	3.23 ± 0.58
acellular collagen I hydrogel	51.08 Pa ± 5.16	37.20 ± 4.35
acellular dense collagen I	474.03 ± 42.5	10.67 ± 0.55
786-O dense collagen I (D1)	503.9 ± 52.5	10.77 ± 0.48
786-O dense collagen I (D21)	348.3 ± 47.2	15.14 ± 1.38
ACHN dense collagen I (D1)	532.9 ± 64.34	12.33 ± 0.55
ACHN dense collagen I (D21)	1253.2 ± 114.2	10.93 ± 0.77
human kidney tumour tissue	1410.10 ± 337.0	10.54 ± 0.43

This work enhances our understanding of the inherent material properties of tissues and *in vitro* tissue models. In particular, it establishes the validity of using the dense collagen I model in investigating the cell driven changes during tumor progression. Unlike commonly used materials such as Matrigel and Geltrex, the dense collagen matrix provides a more biomimetic environment with mechanical properties more like human tissue. This work aims to introduce a comprehensive approach in characterizing the molecular and biophysical behavior of cancer cells in the 3D tissue environment.

The cancer cells used in this study are associated with different genetic mutations, *VHL* for 786-O and *MET* for ACHN cells.^43^ This results in different phenotypic responses and expression of ECM receptors, and consequently diverse patterns of remodelling which is presented in this work. The differences in cell driven matrix remodelling can significantly influence treatment as it can contribute to drug resistance by acting as a physical barrier impeding therapeutic efficacy. Also, the remodelling can indicate the risk of metastasis as changes in the ECM composition can create a more permissive environment for cancer invasion. By investigating the behavior of cells and their matrix, it is possible to elucidate the remodelling pathways and identify targets such as ECM components or enzymes that can enhance drug delivery and efficacy.

ACHN and 786-O cells have been utilized in the literature to study cancer cell migration.^58−60^ One study investigated the role of cell–cell adhesion on tumor invasiveness, showing that less ACHN cells invade and migrate in comparison to 786-O cells and this behavior can be promoted by β-Catenin.^61^ The difference in invasive behavior of the cancer cell lines supports the findings from our work and highlights how the dense collagen model can enable reliable investigation of tumor cell behavior.

The tumoroid model design enables cell driven matrix remodelling to be tracked using molecular and mechanical measurements. The key drivers in the stiffening of the renal tumor microenvironment includes collagen IV over collagen I and the LOX enzyme. The lysyl oxidase (LOX) protein family is known to promote cross-linking of key ECM proteins and is associated with desmoplasia in solid cancers.^62^ In particular, LOX mRNA is significantly overexpressed in patients, correlating with metastatic status and poor survival.^63,64^ The role of LOX in the cancer cultures is cell-type dependent, as the matrix stiffening in ACHN models correlates with upregulation of LOX, however significant reduction in its gene expression was not observed in matrix softening by 786-O cells. In addition, LOX has been shown to enhance renal cancer cell migration and invasion.^65,66^

Matrix remodelling is also closely controlled by expression of MMPs. A family of endopeptidases responsible for the digestion of ECM components, with elevated expression shown to correlate with tumorigenesis and invasion of solid cancers.^67^ MMP9 plays an extensive role in ECM degradation in renal cancers, along with MMP7 and −8. These specific markers are associated with poor prognosis in RCC patients, with roles in regulating cancer invasion and angiogenesis.^68,69^ As the cancer cells remodelled the collagen dense matrix, the expression of MMPs followed similar trends, with the more invasive 786-O cells softening the matrix while upregulating gene expression of MMP7. Although it is not as regularly expressed in healthy kidneys, MMP7 is upregulated in CKD and cancers.^70^

Overexpression of collagen IV and collagen I indicate poor prognosis in ccRCC patient survival^71^ due to the desmoplastic reaction in solid tumor growth. The ECM’s basement membrane is critical in the tumor microenvironment, with collagen IV disruption and expansion driving cancer progression.^72,73^ It also highlights the shift in matrix production during malignancy as it suggests that collagen I is not as directly involved in the matrix stiffening in renal matrices. The increased expression of collagen IV in the tumoroid model further highlights the protein’s structural role as well as its ability to provide a more passive microenvironment for invasive cancer phenotypes.

In addition, Twist1 is a key transcription factor involved in several developmental processes and also plays an important role in tumorigenesis.^74^ Cancer cells will upregulate the marker through upstream pathways such as NF-κB and STAT, to promote EMT and cell migration. The data suggests that Twist1 expression influences matrix remodelling in a cell specific manner, as the trends for gene upregulation with matrix stiffening are only present in 786-O cultures. Although the pathological significance is still unclear, there is potential clinical value in tracking Twist1 expression, as it can indicate tumor aggressiveness in RCC patient tissue samples.^75^

The temporal nature of ECM remodelling during tumor progression is also highlighted using this model, as cancer cells will interact with the 3D collagen architecture and generate forces to aid its proliferation and migration. Our model provides a platform to study the behavior of cancer cells and how they interact with their 3D environment using a natural protein-based *in vitro* ECM like scaffold, enabling us to track the biochemical changes i.e., gene expression and cell migration. In addition to this, we present a method of measuring the mechanical properties of the 3D models that can provide meaningful information regarding how different cancer cells will remodel their matrix. This also holds clinical potential as it can be used to test therapeutics under development to understand drug efficacy and toxicity as well as in patient derived *in vitro* or xenograft models.

Utilizing dense collagen I as a biomimetic material enhances our understanding of the initial pathological events instigated by tumor cells toward the surrounding ECM, shedding light on mechanisms driving malignancy. This holds great potential in identifying key biological markers that can indicate disease stage or identify the role of certain proteins or cytokines in processes such as cell invasion and ECM stiffening. Furthermore, it highlights the potential in personalized approaches to therapeutics as different cell populations can induce significantly different responses within the matrix differentially promoting disease progression. The subtype of RCC can define the mechanisms used by cancer cells to proliferate and metastasize. The model outlined in this work could be used as a platform to investigate and identify markers and mechanisms that RCC patient cells may be using during matrix remodelling. This could enable more tailored therapeutic pathways such as MMP inhibitors, integrin inhibitors or antifibrotic agents such as pirfenidone.^26^

Although this work demonstrates the benefits of using a 3D *in vitro* system to study cancer cell behavior, there are limitations to the current work that can be addressed in future research. The kidney is populated with multiple important cell populations and this needs to be reflected in the system, for example the introduction of stromal cells in the compartmentalized model may elucidate a better understanding of how cancer cells remodel their ECM. In addition, factors such as vascular networks and ECM composition are critical in tumor development. By incorporating endothelial cells and other basement membrane proteins, we can achieve a more biomimetic system. These elements have been incorporated into the collagen model for other diseases^13,48,76,77^ and would be beneficial in our future research.

## Data Availability

The raw/processed data can be found on the manuscript/supplementary files. Any other information is publicly available through the online repository.

## Supporting Information Available

Additional experimental details for IF staining and primer sequences for qPCR primers. Figure 1: Further information on elastic moduli readings of biomaterials. Figures 2 and 3: Proliferation assays and images of cells in various conditions. Figures 4–7: Elastic moduli readings and full gene expression panels for cancer cell 3D cultures (PDF)

## Notes

All methods were carried out in accordance with relevant guidelines and regulations. Patient samples were obtained, with informed consent, from patients with renal tumors through the Tissue Access for Patient Benefit initiative at The Royal Free Hospital, London, UK. The ethics was approved by the University College London Royal Free Hospital BioBank Ethical Review Committee; Research Ethics Committee Reference number (21/WA/0388).

## References

[ref1] Frantz C.; Stewart K. M.; Weaver V. M. The extracellular matrix at a glance. J. Cell Sci. 2010, 123 (24), 4195–4200. 10.1242/jcs.023820. 21123617 PMC2995612

[ref2] Bülow R. D.; Boor P. Extracellular Matrix in Kidney Fibrosis: More Than Just a Scaffold. J. Histochem. Cytochem. 2019, 67 (9), 643–661. 10.1369/0022155419849388. 31116062 PMC6713975

[ref3] Cox T. R. The matrix in cancer. Nat. Rev. Cancer 2021, 21 (4), 217–238. 10.1038/s41568-020-00329-7. 33589810

[ref4] Majo S.; Courtois S.; Souleyreau W.; Bikfalvi A.; Auguste P. Impact of Extracellular Matrix Components to Renal Cell Carcinoma Behavior. Front. Oncol. 2020, 10, 625 10.3389/fonc.2020.00625. 32411604 PMC7198871

[ref5] Handorf A. M.; Zhou Y.; Halanski M. A.; Li W. J. Tissue stiffness dictates development, homeostasis, and disease progression. Organogenesis 2015, 11 (1), 1–15. 10.1080/15476278.2015.1019687. 25915734 PMC4594591

[ref6] Klingberg F.; Hinz B.; White E. S. The myofibroblast matrix: implications for tissue repair and fibrosis. J. Pathol. 2013, 229 (2), 298–309. 10.1002/path.4104. 22996908 PMC4005341

[ref7] Falke L. L.; Gholizadeh S.; Goldschmeding R.; Kok R. J.; Nguyen T. Q. Diverse origins of the myofibroblast—implications for kidney fibrosis. Nat. Rev. Nephrol. 2015, 11 (4), 233–244. 10.1038/nrneph.2014.246. 25584804

[ref8] Liu Y. Cellular and molecular mechanisms of renal fibrosis. Nat. Rev. Nephrol. 2011, 7 (12), 684–696. 10.1038/nrneph.2011.149. 22009250 PMC4520424

[ref9] Kong W.; Lyu C.; Liao H.; Du Y. Collagen crosslinking: effect on structure, mechanics and fibrosis progression. Biomed. Mater. 2021, 16 (6), 062005 10.1088/1748-605X/ac2b79. 34587604

[ref10] Tatsukawa H.; Hitomi K. Role of Transglutaminase 2 in Cell Death, Survival, and Fibrosis. Cells 2021, 10 (7), 1842 10.3390/cells10071842. 34360011 PMC8307792

[ref11] Laczko R.; Csiszar K. Lysyl Oxidase (LOX): Functional Contributions to Signaling Pathways. Biomolecules 2020, 10 (8), 1093 10.3390/biom10081093. 32708046 PMC7465975

[ref12] Levental I.; Georges P. C.; Janmey P. A. Soft biological materials and their impact on cell function. Soft Matter 2007, 3 (3), 299–306. 10.1039/B610522J. 32900146

[ref13] Pape J.; Magdeldin T.; Stamati K.; Nyga A.; Loizidou M.; Emberton M.; Cheema U. Cancer-associated fibroblasts mediate cancer progression and remodel the tumouroid stroma. Br. J. Cancer 2020, 123 (7), 1178–1190. 10.1038/s41416-020-0973-9. 32641866 PMC7524802

[ref14] Capitanio U.; Bensalah K.; Bex A.; Boorjian S. A.; Bray F.; Coleman J.; Gore J. L.; Sun M.; Wood C.; Russo P. Epidemiology of Renal Cell Carcinoma. Eur. Urol. 2019, 75 (1), 74–84. 10.1016/j.eururo.2018.08.036. 30243799 PMC8397918

[ref15] Delahunt B.; Eble J. N.; Samaratunga H.; Thunders M.; Yaxley J. W.; Egevad L. Staging of renal cell carcinoma: current progress and potential advances. Pathology 2021, 53 (1), 120–128. 10.1016/j.pathol.2020.08.007. 33121821

[ref16] Bahadoram S.; Davoodi M.; Hassanzadeh S.; Bahadoram M.; Barahman M.; Mafakher L. Renal cell carcinoma: an overview of the epidemiology, diagnosis, and treatment. G Ital Nefrol. 2022, 39 (3), 2022. 35819037

[ref17] Zhang S.; Fang T.; He Y.; Feng W.; Yu Z.; Zheng Y.; Zhang C.; Hu S.; Liu Z.; Liu J.; Yu J.; Zhang H.; He A.; Gong Y.; He Z.; Yang K.; Xi Z.; Yu W.; Zhou L.; Yao L.; Yue S. VHL mutation drives human clear cell renal cell carcinoma progression through PI3K/AKT-dependent cholesteryl ester accumulation. EBioMedicine 2024, 103, 105070 10.1016/j.ebiom.2024.105070. 38564827 PMC10999658

[ref18] Shenoy N.; Pagliaro L. Sequential pathogenesis of metastatic VHL mutant clear cell renal cell carcinoma: putting it together with a translational perspective. Ann. Oncol. 2016, 27 (9), 1685–1695. 10.1093/annonc/mdw241. 27329246

[ref19] Ohh M.; Yauch R. L.; Lonergan K. M.; Whaley J. M.; Stemmer-Rachamimov A. O.; Louis D. N.; Gavin B. J.; Kley N.; Kaelin W. G. Jr.; Iliopoulos O. The von Hippel-Lindau tumor suppressor protein is required for proper assembly of an extracellular fibronectin matrix. Mol. Cell 1998, 1 (7), 959–968. 10.1016/S1097-2765(00)80096-9. 9651579

[ref20] Kurban G.; Duplan E.; Ramlal N.; Hudon V.; Sado Y.; Ninomiya Y.; Pause A. Collagen matrix assembly is driven by the interaction of von Hippel-Lindau tumor suppressor protein with hydroxylated collagen IV alpha 2. Oncogene 2008, 27 (7), 1004–1012. 10.1038/sj.onc.1210709. 17700531

[ref21] Gibney G. T.; Aziz S. A.; Camp R. L.; Conrad P.; Schwartz B. E.; Chen C. R.; Kelly W. K.; Kluger H. M. c-Met is a prognostic marker and potential therapeutic target in clear cell renal cell carcinoma. Ann. Oncol. 2013, 24 (2), 343–349. 10.1093/annonc/mds463. 23022995 PMC3551486

[ref22] Kim J. H.; Kim B. J.; Kim H. S. Clinicopathological impacts of high c-Met expression in renal cell carcinoma: a meta-analysis and review. Oncotarget 2017, 8 (43), 75478–75487. 10.18632/oncotarget.20796. 29088883 PMC5650438

[ref23] Paszek M. J.; Zahir N.; Johnson K. R.; Lakins J. N.; Rozenberg G. I.; Gefen A.; Reinhart-King C. A.; Margulies S. S.; Dembo M.; Boettiger D.; Hammer D. A.; Weaver V. M. Tensional homeostasis and the malignant phenotype. Cancer Cell 2005, 8 (3), 241–254. 10.1016/j.ccr.2005.08.010. 16169468

[ref24] Micalet A.; Pape J.; Bakkalci D.; Javanmardi Y.; Hall C.; Cheema U.; Moeendarbary E. Evaluating the Impact of a Biomimetic Mechanical Environment on Cancer Invasion and Matrix Remodeling. Adv. Healthcare Mater. 2023, 12 (14), e2201749 10.1002/adhm.202201749. PMC11468596 36333907

[ref25] Mikami S.; Oya M.; Mizuno R.; Kosaka T.; Katsube K.-i.; Okada Y. Invasion and metastasis of renal cell carcinoma. Med. Mol. Morphol. 2014, 47 (2), 63–67. 10.1007/s00795-013-0064-6. 24213520

[ref26] Winkler J.; Abisoye-Ogunniyan A.; Metcalf K. J.; Werb Z. Concepts of extracellular matrix remodelling in tumour progression and metastasis. Nat. Commun. 2020, 11 (1), 5120 10.1038/s41467-020-18794-x. 33037194 PMC7547708

[ref27] Kechagia J. Z.; Ivaska J.; Roca-Cusachs P. Integrins as biomechanical sensors of the microenvironment. Nat. Rev. Mol. Cell Biol. 2019, 20 (8), 457–473. 10.1038/s41580-019-0134-2. 31182865

[ref28] Tian H.; Shi H.; Yu J.; Ge S.; Ruan J. Biophysics Role and Biomimetic Culture Systems of ECM Stiffness in Cancer EMT. Glob. Chall. 2022, 6 (6), 2100094 10.1002/gch2.202100094. 35712024 PMC9189138

[ref29] Guimarães C. F.; Gasperini L.; Marques A. P.; Reis R. L. The stiffness of living tissues and its implications for tissue engineering. Nat. Rev. Mater. 2020, 5 (5), 351–370. 10.1038/s41578-019-0169-1.

[ref30] Inci M. F.; Kalayci T. O.; Tan S.; Karasu S.; Albayrak E.; Cakir V.; Ocal I.; Ozkan F. Diagnostic value of strain elastography for differentiation between renal cell carcinoma and transitional cell carcinoma of kidney. Abdom. Radiol. 2016, 41 (6), 1152–1159. 10.1007/s00261-016-0658-2. 26880174

[ref31] Leong S. S.; Wong J. H. D.; Md Shah M. N.; Vijayananthan A.; Jalalonmuhali M.; Chow T. K.; Sharif N. H. M.; Ng K. H. Shear wave elastography accurately detects chronic changes in renal histopathology. Nephrology 2021, 26 (1), 38–45. 10.1111/nep.13805. 33058334

[ref32] Rouvière O.; Souchon R.; Pagnoux G.; Ménager J.-M.; Chapelon J.-Y. Magnetic resonance elastography of the kidneys: Feasibility and reproducibility in young healthy adults. J. Magn. Reson. Imaging 2011, 34 (4), 880–886. 10.1002/jmri.22670. 21769970 PMC3176985

[ref33] Cirka H. A.; Koehler S. A.; Farr W. W.; Billiar K. L. Eccentric rheometry for viscoelastic characterization of small, soft, anisotropic, and irregularly shaped biopolymer gels and tissue biopsies. Ann. Biomed. Eng. 2012, 40 (8), 1654–1665. 10.1007/s10439-012-0532-5. 22361829 PMC3896300

[ref34] Miron-Mendoza M.; Seemann J.; Grinnell F. The differential regulation of cell motile activity through matrix stiffness and porosity in three dimensional collagen matrices. Biomaterials 2010, 31 (25), 6425–6435. 10.1016/j.biomaterials.2010.04.064. 20537378 PMC2900504

[ref35] Piechocka I. K.; van Oosten A. S.; Breuls R. G.; Koenderink G. H. Rheology of heterotypic collagen networks. Biomacromolecules 2011, 12 (7), 2797–2805. 10.1021/bm200553x. 21671664

[ref36] van Oosten A. S. G.; Vahabi M.; Licup A. J.; Sharma A.; Galie P. A.; MacKintosh F. C.; Janmey P. A. Uncoupling shear and uniaxial elastic moduli of semiflexible biopolymer networks: compression-softening and stretch-stiffening. Sci. Rep. 2016, 6 (1), 19270 10.1038/srep19270. 26758452 PMC4725936

[ref37] Valero C.; Amaveda H.; Mora M.; García-Aznar J. M. Combined experimental and computational characterization of crosslinked collagen-based hydrogels. PLoS One 2018, 13 (4), e0195820 10.1371/journal.pone.0195820. 29664953 PMC5903660

[ref38] Achilli M.; Mantovani D. Tailoring Mechanical Properties of Collagen-Based Scaffolds for Vascular Tissue Engineering: The Effects of pH, Temperature and Ionic Strength on Gelation. Polymers 2010, 2 (4), 664–680. 10.3390/polym2040664.

[ref39] Chaudhuri O.; Cooper-White J.; Janmey P. A.; Mooney D. J.; Shenoy V. B. Effects of extracellular matrix viscoelasticity on cellular behaviour. Nature 2020, 584 (7822), 535–546. 10.1038/s41586-020-2612-2. 32848221 PMC7676152

[ref40] Storm C.; Pastore J. J.; MacKintosh F. C.; Lubensky T. C.; Janmey P. A. Nonlinear elasticity in biological gels. Nature 2005, 435 (7039), 191–194. 10.1038/nature03521. 15889088

[ref41] Redova M.; Poprach A.; Besse A.; Iliev R.; Nekvindova J.; Lakomy R.; Radova L.; Svoboda M.; Dolezel J.; Vyzula R.; Slaby O. MiR-210 expression in tumor tissue and in vitro effects of its silencing in renal cell carcinoma. Tumor Biol. 2013, 34 (1), 481–491. 10.1007/s13277-012-0573-2. 23150176

[ref42] Brodaczewska K. K.; Szczylik C.; Fiedorowicz M.; Porta C.; Czarnecka A. M. Choosing the right cell line for renal cell cancer research. Mol. Cancer 2016, 15 (1), 83 10.1186/s12943-016-0565-8. 27993170 PMC5168717

[ref43] Shapiro D. D.; Virumbrales-Muñoz M.; Beebe D. J.; Abel E. J. Models of Renal Cell Carcinoma Used to Investigate Molecular Mechanisms and Develop New Therapeutics. Front. Oncol. 2022, 12, 871252 10.3389/fonc.2022.871252. 35463327 PMC9022005

[ref44] David G. Chapter 35 - Collagen-based 3D structures—versatile, efficient materials for biomedical applications. In Biopolymer-Based Formulations; Pal K., Ed.; Elsevier, 2020; pp 881–906.

[ref45] Moroni L.; Burdick J. A.; Highley C.; Lee S. J.; Morimoto Y.; Takeuchi S.; Yoo J. J. Biofabrication strategies for 3D in vitro models and regenerative medicine. Nat. Rev. Mater. 2018, 3 (5), 21–37. 10.1038/s41578-018-0006-y. 31223488 PMC6586020

[ref46] Kamranpour N. O.; Miri A. K.; James-Bhasin M.; Nazhat S. N. A gel aspiration-ejection system for the controlled production and delivery of injectable dense collagen scaffolds. Biofabrication 2016, 8 (1), 015018 10.1088/1758-5090/8/1/015018. 27003606

[ref47] Bakkalci D.; Al-Badri G.; Yang W.; Nam A.; Liang Y.; Khurram S. A.; Heavey S.; Fedele S.; Cheema U. Spatial transcriptomic interrogation of the tumour-stroma boundary in a 3D engineered model of ameloblastoma. Mater. Today Bio 2024, 24, 100923 10.1016/j.mtbio.2023.100923. PMC10788620 38226014

[ref48] Micalet A.; Tappouni L. J.; Peszko K.; Karagianni D.; Lam A.; Counsell J. R.; Quezada S. A.; Moeendarbary E.; Cheema U. Urokinase-type plasminogen activator (uPA) regulates invasion and matrix remodelling in colorectal cancer. Matrix Biol. Plus 2023, 19–20, 100137 10.1016/j.mbplus.2023.100137. PMC10667746 38020586

[ref49] Pape J.; Stamati K.; Al Hosni R.; Uchegbu I. F.; Schatzlein A. G.; Loizidou M.; Emberton M.; Cheema U. Tissue-Engineering the Fibrous Pancreatic Tumour Stroma Capsule in 3D Tumouroids to Demonstrate Paclitaxel Response. Int. J. Mol. Sci. 2021, 22 (8), 4289 10.3390/ijms22084289. 33924238 PMC8074746

[ref50] Rio D. C.; Ares M.; Hannon G. J.; Nilsen T. W. Purification of RNA using TRIzol (TRI reagent). Cold Spring Harb. Protoc. 2010, 2010 (6), pdb.prot5439 10.1101/pdb.prot5439. 20516177

[ref51] Wei S. C.; Fattet L.; Tsai J. H.; Guo Y.; Pai V. H.; Majeski H. E.; Chen A. C.; Sah R. L.; Taylor S. S.; Engler A. J.; Yang J. Matrix stiffness drives epithelial-mesenchymal transition and tumour metastasis through a TWIST1-G3BP2 mechanotransduction pathway. Nat. Cell Biol. 2015, 17 (5), 678–688. 10.1038/ncb3157. 25893917 PMC4452027

[ref52] Kim K. P.; Williams C. E.; Lemmon C. A. Cell–Matrix Interactions in Renal Fibrosis. Kidney Dial. 2022, 2 (4), 607–624. 10.3390/kidneydial2040055. 37033194 PMC10081509

[ref53] Karimi A.; Shojaei A. Measurement of the Mechanical Properties of the Human Kidney. IRBM 2017, 38 (5), 292–297. 10.1016/j.irbm.2017.08.001.

[ref54] Leong S. S.; Wong J. H. D.; Md Shah M. N.; Vijayananthan A.; Jalalonmuhali M.; Mohd Sharif N. H.; Abas N. K.; Ng K. H. Stiffness and Anisotropy Effect on Shear Wave Elastography: A Phantom and in Vivo Renal Study. Ultrasound Med. Biol. 2020, 46 (1), 34–45. 10.1016/j.ultrasmedbio.2019.08.011. 31594681

[ref55] Bensamoun S. F.; Robert L.; Leclerc G. E.; Debernard L.; Charleux F. Stiffness imaging of the kidney and adjacent abdominal tissues measured simultaneously using magnetic resonance elastography. Clin. Imaging 2011, 35 (4), 284–287. 10.1016/j.clinimag.2010.07.009. 21724121

[ref56] Samir A. E.; Allegretti A. S.; Zhu Q.; Dhyani M.; Anvari A.; Sullivan D. A.; Trottier C. A.; Dougherty S.; Williams W. W.; Babitt J. L.; Wenger J.; Thadhani R. I.; Lin H. Y. Shear wave elastography in chronic kidney disease: a pilot experience in native kidneys. BMC Nephrol. 2015, 16, 119 10.1186/s12882-015-0120-7. 26227484 PMC4521488

[ref57] Castro A. P. G.; Laity P.; Shariatzadeh M.; Wittkowske C.; Holland C.; Lacroix D. Combined numerical and experimental biomechanical characterization of soft collagen hydrogel substrate. J. Mater. Sci. Mater. Med. 2016, 27 (4), 79 10.1007/s10856-016-5688-3. 26914710 PMC4767858

[ref58] Ta L.; Xuan C.; Xing N.; Zhu X. COP1 is downregulated in renal cell carcinoma (RCC) and inhibits the migration of RCC ACHN cells in vitro. Mol. Med. Rep. 2016, 14 (2), 1371–1378. 10.3892/mmr.2016.5373. 27278120

[ref59] Liu L. J.; Yu J. J.; Xu X. L. MicroRNA-93 inhibits apoptosis and promotes proliferation, invasion and migration of renal cell carcinoma ACHN cells via the TGF-β/Smad signaling pathway by targeting RUNX3. Am. J. Transl. Res. 2017, 9 (7), 3499–3513. 28804566 PMC5527264

[ref60] Zhao C.-X.; Luo C.-L.; Wu X.-H. Hypoxia promotes 786-O cells invasiveness and resistance to sorafenib via HIF-2α/COX-2. Med. Oncol. 2015, 32 (1), 419 10.1007/s12032-014-0419-4. 25487445

[ref61] Yang C.-m.; Ji S.; Li Y.; Fu L.-y.; Jiang T.; Meng F.-d. β-Catenin promotes cell proliferation, migration, and invasion but induces apoptosis in renal cell carcinoma. OncoTargets Ther. 2017, 10, 711–724. 10.2147/OTT.S117933. PMC5328321 28260916

[ref62] Barker H. E.; Cox T. R.; Erler J. T. The rationale for targeting the LOX family in cancer. Nat. Rev. Cancer 2012, 12 (8), 540–552. 10.1038/nrc3319. 22810810

[ref63] Lin S.; Zheng L.; Lu Y.; Xia Q.; Zhou P.; Liu Z. Comprehensive analysis on the expression levels and prognostic values of LOX family genes in kidney renal clear cell carcinoma. Cancer Med. 2020, 9 (22), 8624–8638. 10.1002/cam4.3472. 32970930 PMC7666732

[ref64] Di Stefano V.; Torsello B.; Bianchi C.; Cifola I.; Mangano E.; Bovo G.; Cassina V.; De Marco S.; Corti R.; Meregalli C.; Bombelli S.; Viganò P.; Battaglia C.; Strada G.; Perego R. A. Major Action of Endogenous Lysyl Oxidase in Clear Cell Renal Cell Carcinoma Progression and Collagen Stiffness Revealed by Primary Cell Cultures. Am. J. Pathol. 2016, 186 (9), 2473–2485. 10.1016/j.ajpath.2016.05.019. 27449199

[ref65] Kurozumi A.; Kato M.; Goto Y.; Matsushita R.; Nishikawa R.; Okato A.; Fukumoto I.; Ichikawa T.; Seki N. Regulation of the collagen cross-linking enzymes LOXL2 and PLOD2 by tumor-suppressive microRNA-26a/b in renal cell carcinoma. Int. J. Oncol. 2016, 48 (5), 1837–1846. 10.3892/ijo.2016.3440. 26983694 PMC4809659

[ref66] Hase H.; Jingushi K.; Ueda Y.; Kitae K.; Egawa H.; Ohshio I.; Kawakami R.; Kashiwagi Y.; Tsukada Y.; Kobayashi T.; Nakata W.; Fujita K.; Uemura M.; Nonomura N.; Tsujikawa K. LOXL2 status correlates with tumor stage and regulates integrin levels to promote tumor progression in ccRCC. Mol. Cancer Res. 2014, 12 (12), 1807–1817. 10.1158/1541-7786.MCR-14-0233. 25092917

[ref67] Hashmi F.; Mollapour M.; Bratslavsky G.; Bourboulia D. MMPs, tyrosine kinase signaling and extracellular matrix proteolysis in kidney cancer. Urol. Oncol. 2021, 39 (6), 316–321. 10.1016/j.urolonc.2020.04.034. 32487351 PMC9533233

[ref68] Kushlinskii N. E.; Gershtein E. S.; Alferov A. A.; Bezhanova S. D.; Mushtenko V. V.; Pushkar D. Y.; Matveev V. B.; Stilidi I. S. Prognostic Role of Matrix Metalloproteinases 2, 7, 8, 9 and Their Type 1 Tissue Inhibitor in Blood Serum of Patients with Kidney Cancer. Bull. Exp. Biol. Med. 2020, 168 (5), 673–676. 10.1007/s10517-020-04778-w. 32248449

[ref69] Miyata Y.; Iwata T.; Ohba K.; Kanda S.; Nishikido M.; Kanetake H. Expression of Matrix Metalloproteinase-7 on Cancer Cells and Tissue Endothelial Cells in Renal Cell Carcinoma: Prognostic Implications and Clinical Significance for Invasion and Metastasis. Clin. Cancer Res. 2006, 12 (23), 6998–7003. 10.1158/1078-0432.CCR-06-1626. 17145820

[ref70] Liu Z.; Tan R. J.; Liu Y. The Many Faces of Matrix Metalloproteinase-7 in Kidney Diseases. Biomolecules 2020, 10 (6), 960 10.3390/biom10060960. 32630493 PMC7356035

[ref71] Majo S.; Courtois S.; Souleyreau W.; Bikfalvi A.; Auguste P. Impact of Extracellular Matrix Components to Renal Cell Carcinoma Behavior. Front. Oncol. 2020, 10, 625 10.3389/fonc.2020.00625. 32411604 PMC7198871

[ref72] Miyake M.; Hori S.; Morizawa Y.; Tatsumi Y.; Toritsuka M.; Ohnishi S.; Shimada K.; Furuya H.; Khadka V. S.; Deng Y.; Ohnishi K.; Iida K.; Gotoh D.; Nakai Y.; Inoue T.; Anai S.; Torimoto K.; Aoki K.; Tanaka N.; Konishi N.; Fujimoto K. Collagen type IV alpha 1 (COL4A1) and collagen type XIII alpha 1 (COL13A1) produced in cancer cells promote tumor budding at the invasion front in human urothelial carcinoma of the bladder. Oncotarget 2017, 8 (22), 36099–36114. 10.18632/oncotarget.16432. 28415608 PMC5482641

[ref73] Brenner W.; Groß S.; Steinbach F.; Horn S.; Hohenfellner R.; Thüroff J. W. Differential inhibition of renal cancer cell invasion mediated by fibronectin, collagen IV and laminin. Cancer Lett. 2000, 155 (2), 199–205. 10.1016/S0304-3835(00)00429-8. 10822136

[ref74] Qin Q.; Xu Y.; He T.; Qin C.; Xu J. Normal and disease-related biological functions of Twist1 and underlying molecular mechanisms. Cell Res. 2012, 22 (1), 90–106. 10.1038/cr.2011.144. 21876555 PMC3351934

[ref75] Ohba K.; Miyata Y.; Matsuo T.; Asai A.; Mitsunari K.; Shida Y.; Kanda S.; Sakai H. High expression of Twist is associated with tumor aggressiveness and poor prognosis in patients with renal cell carcinoma. Int. J. Clin. Exp. Pathol. 2014, 7 (6), 3158–3165. 25031735 PMC4097249

[ref76] Stamati K.; Priestley J. V.; Mudera V.; Cheema U. Laminin promotes vascular network formation in 3D in vitro collagen scaffolds by regulating VEGF uptake. Exp. Cell Res. 2014, 327 (1), 68–77. 10.1016/j.yexcr.2014.05.012. 24907654 PMC4155934

[ref77] Magdeldin T.; López-Dávila V.; Pape J.; Cameron G. W. W.; Emberton M.; Loizidou M.; Cheema U. Engineering a vascularised 3D in vitro model of cancer progression. Sci. Rep. 2017, 7 (1), 44045 10.1038/srep44045. 28276469 PMC5343474

