# Supplementary material for: Dense Collagen I as a Biomimetic Material to Track Matrix Remodelling in Renal Carcinomas
Source: ACS Omega. 2024 Sep 28;9(40):41419–32. doi: 10.1021/acsomega.4c04442 (PMC11465592; doi:10.1021/acsomega.4c04442)
Supplement: Supplementary file 1 — ao4c04442_si_001.pdf [file ao4c04442_si_001.pdf]

Dense Collagen I as a Biomimetic Material to Track Matrix Remodelling in Renal Carcinomas

Anuja Upadhyay*[1], Deniz Bakkalci[1], Auxtine Micalet[1,3], Matt Butler[2], Marianne Bergin[2], Emad Moeendarbary[3], Marilena Loizidou[4**], Umber Cheema[1**]

[1] UCL Centre for 3D Models of Health and Disease, Division of Surgery and Interventional Science, University College London, Charles Bell House, 43-45 Foley Street, W1W 7TS, London, United Kingdom.

[2]UCB Pharma, 216 Bath Road, SL1 3WE, Slough, United Kingdom.

[3] Department of Mechanical Engineering, Roberts Building, University College London, WC1E 6BT, London, United Kingdom.

[4] University College London, Division of Surgery and Interventional Science, Royal Free Campus, Rowland Hill Street, NW3 2PF, London, United Kingdom.

(**)These authors share senior authorship (Marilena Loizidou and Umber Cheema)

Corresponding author (*): Anuja Upadhyay: anuja.upadhyay.19@ucl.ac.uk

Keywords: Tumouroid, Collagen I, Kidney tissue stiffness, Rheology, 3D tissue model, Extracellular Matrix

Contents

Supplementary Table 1 (Table S1). 3
Supplementary Table 2 (Table S2). 4
Supplementary Figure 1..5
Supplementary Figure 2..6
Supplementary Figure 3..7
....8
Supplementary Figure 4..9
Supplementary Figure 5..11
Supplementary Figure 6..13
Supplementary Figure 7..15

Supplementary Table 1 (Table S1).

Gene Name	Forward Primer Sequence	Reverse Primer Sequence
MMP7	F’- ATG AAC GCT GGA CGG ATG GTA G-3’	5’- GGG ATC TCC ATT TCC ATA GGT TGG-3’
MMP8	5’- AAG CAC ACC CAA ACC CTG TGA C-3’	5’-TCG ACT CTT TGT AGC TGA GGA TGC-3’
MMP9	5’- CCC GGA CCA AGG ATA CAG TTT G-3’<br>5’-CAG TCC ACC CTT GTG CTC TTC C-3’	5’-TGC CAT TCA CGT CGT CCT TAT G-3’<br>5’-TTC GAC TCT CCA CGC ATC TCT G-3’
VIM (Vimentin)	5’- TCT CTG GCA CGT CTT GAC CTT G-3’	5’-CGA TTT GGA CAT GCT GTT CCT G-3’
VEGFα	5’- GCC TTG CCT TGC TGC TCT AC-3’	5’- GAA GAT GTC CAC CAG GGT CTC G-3’
COL4 (Collagen IV)	5’-AAG TTC AGC ACA ATG CCC TTC C-3’	5’- GCC TCA CAC ACA GCA CAC CTA C-3’
EGFR	5’-GGC CGA CAG CTA TGA GAT GGA G-3’	5’-AGA TCG CCA CTG ATG GAG GTG-3’
TWIST1	5’- GAC TCC AAG ATG GCA AGC TG-3’	5’-CTA GTG GGA CGC GGA CAT GG-3’
LOX OR LOXL2	5’-ACA GGG TGC TGC TCA GAT TTC C-3’<br>5’-GGA GGA TGT CGG TGT GGT GTG-3’	5’- AAC TTG CTT TGT GGC CTT CAG C-3’<br>5’- TTG CGG TAG GTT GAG AGG ATG G-3’

Supplementary Table 1: List of all qPCR primers used in the experiments, with gene name and forward and reverse sequences.

Supplementary Table 2 (Table S2).

Antibody	Catalogue Number	Dilution
Cytokeratin-8 Primary Antibody	AB9023	1:200
Alexa Flour 488	A11001	1:500
Phalloidin- Alexa Flour 568	A12380	1:200

Supplementary Table 2: List of all primary and secondary antibodies used in immunofluorescence imaging, with experimental dilutions and catalogue numbers.

Supplementary Figure 1

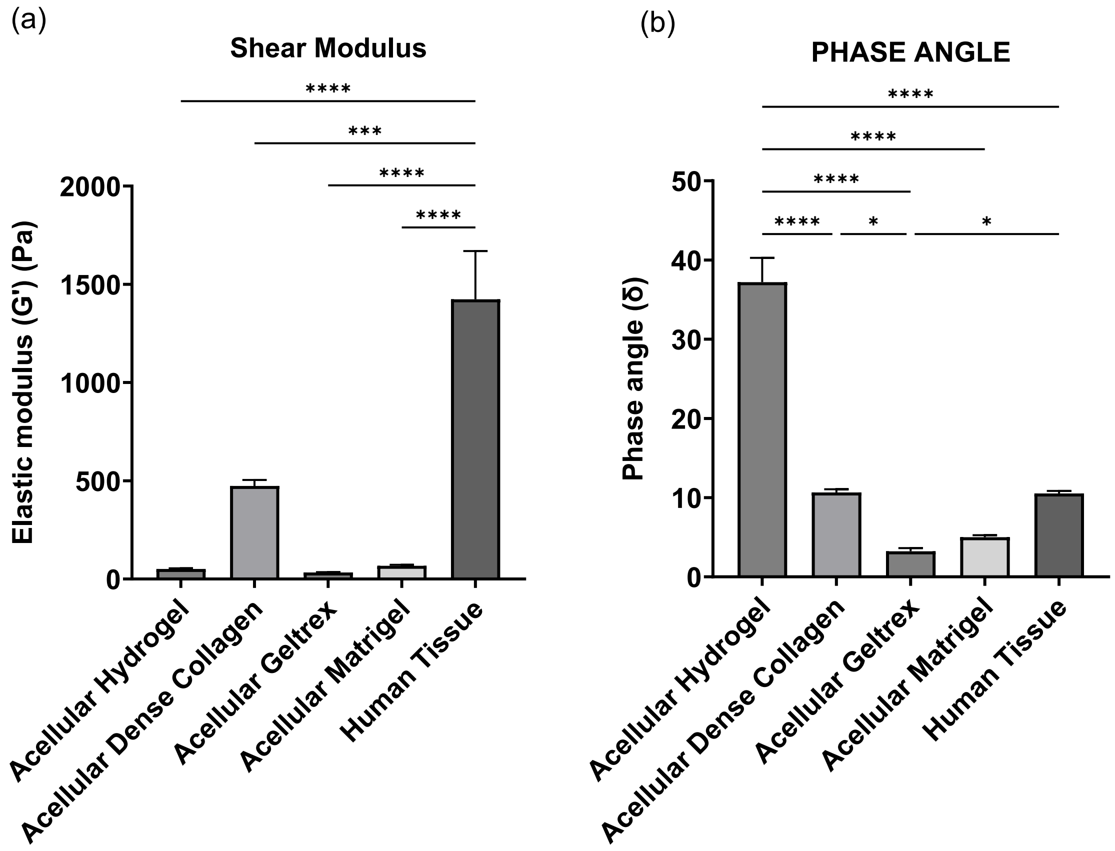

Supp. Figure 1. Rheological data on bioengineered models and human tissue. (a) Shear modulus and (b) phase angle graphed from all measurements. N=3 for all samples and One-Way ANOVA and Tukey's multiple comparisons tests were conducted. (a) p-values ****p<0.0001, ***p<0.0009 (b) ****p<0.0001, *p<0.02. Error bars represent SEM.

Supplementary Figure 2

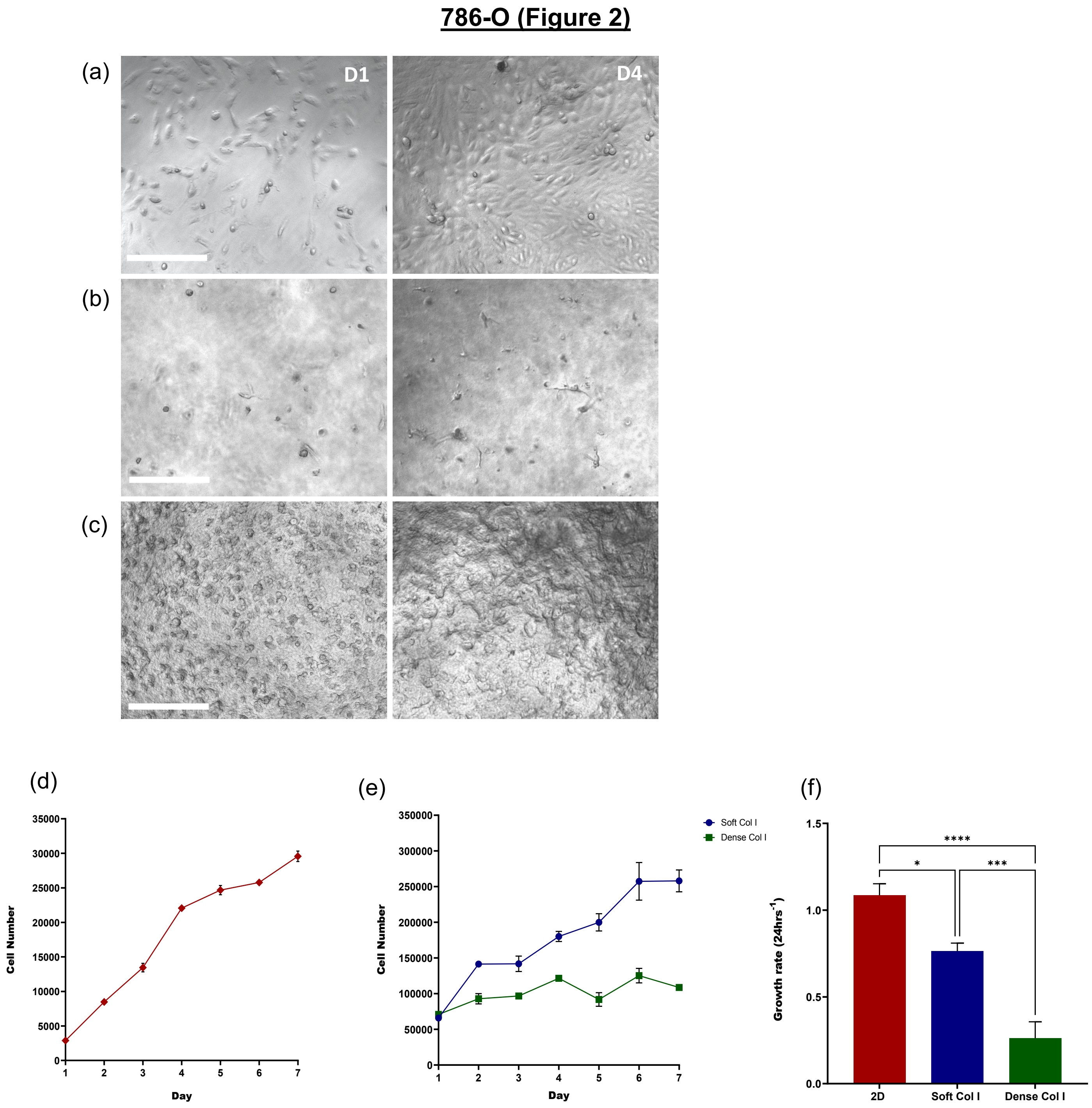

Supp. Figure 2. Proliferation and morphology of 786-O. Light microscopy images in 2D **(a)**, soft collagen I **(b)** and dense collagen I **(c)** gel cultures at day 1 and 4, scale bar 500 µm.

(d) Average cell number of 786-O cultures in 2D 96-well cultures (n=5) over 7 days, error bars represent standard error of mean (SEM). **(e)** Average cell number of 786-O 3D collagen I cultures over 7 days, error bars represent standard error of mean (SEM). **(f)** Average growth rate over 24 hours in 786-O cells in different cultures (n=5, for each condition). Error bars represent SEM. Shapiro- Wilks test and One-Way ANOVA and Tukey's multiple comparisons. p-values *p<0.01, ***p<0.001, ****p<0.0001. 2D seeding density was 5000 cells compared to all 3D cultures at 50,000 cells.

Supplementary Figure 3

ACHN (Figure 3)

(a)
(b)
(c)

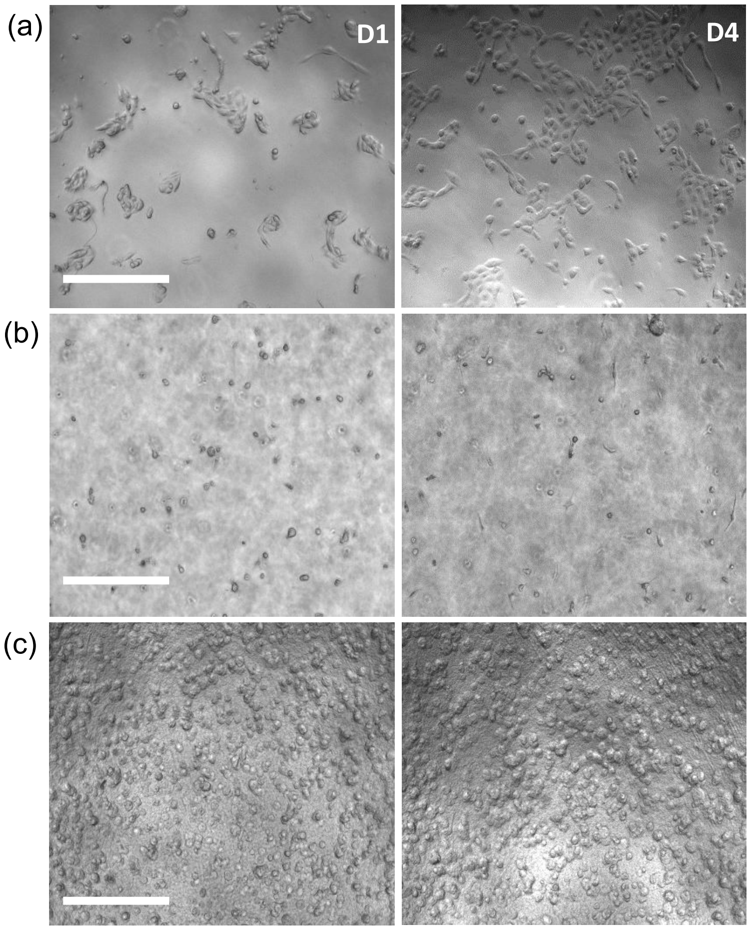

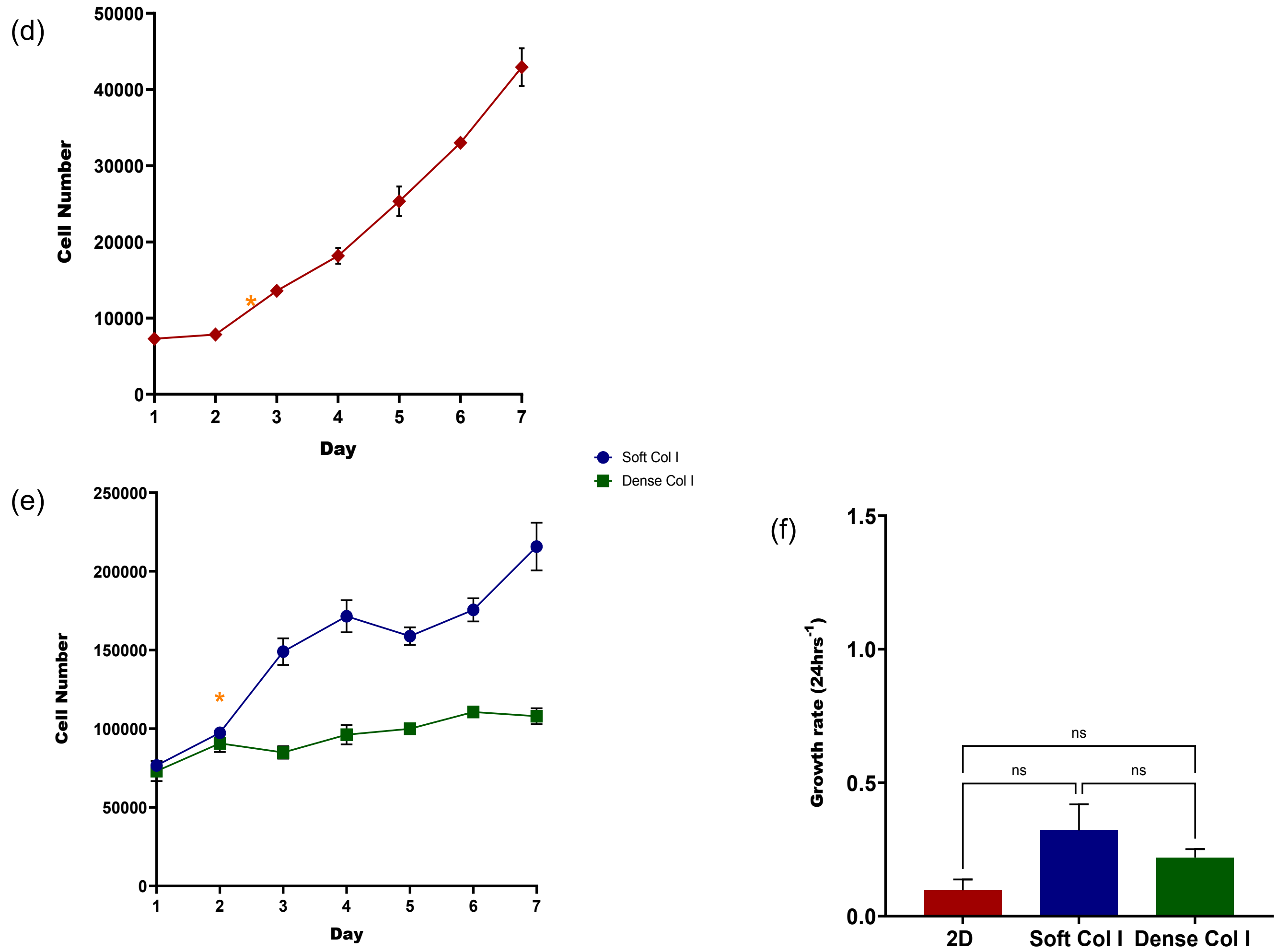

Supp. Figure 3. Proliferation and morphology of ACHN. Light microscopy images in 2D **(a)**, soft collagen I **(b)** and dense collagen I **(c)** gel cultures at day 1 and 4, scale bar 500 µm. **(d)** Average cell number in ACHN cultures in 2D 96-well cultures (n=5) over 7 days, error bars represent standard error of mean (SEM). Orange asterisk indicates point when spheroid formation was first identified in the cultures.

(e) Average cell number of ACHN 3D collagen I cultures over 7 days, error bars represent standard error of mean (SEM). Orange asterisk indicates point when spheroid formation was first identified in the cultures. **(f)** Average growth rate over 24 hours in ACHN cells in different cultures (n=5, for each condition). Error bars represent SEM. Shapiro- Wilks test and One-Way ANOVA and Tukey's multiple comparisons (ns, non-significant). 2D seeding density was 5000 cells compared to all 3D cultures at 50,000 cells.

Supplementary Figure 4

ACHN (Figure 4)

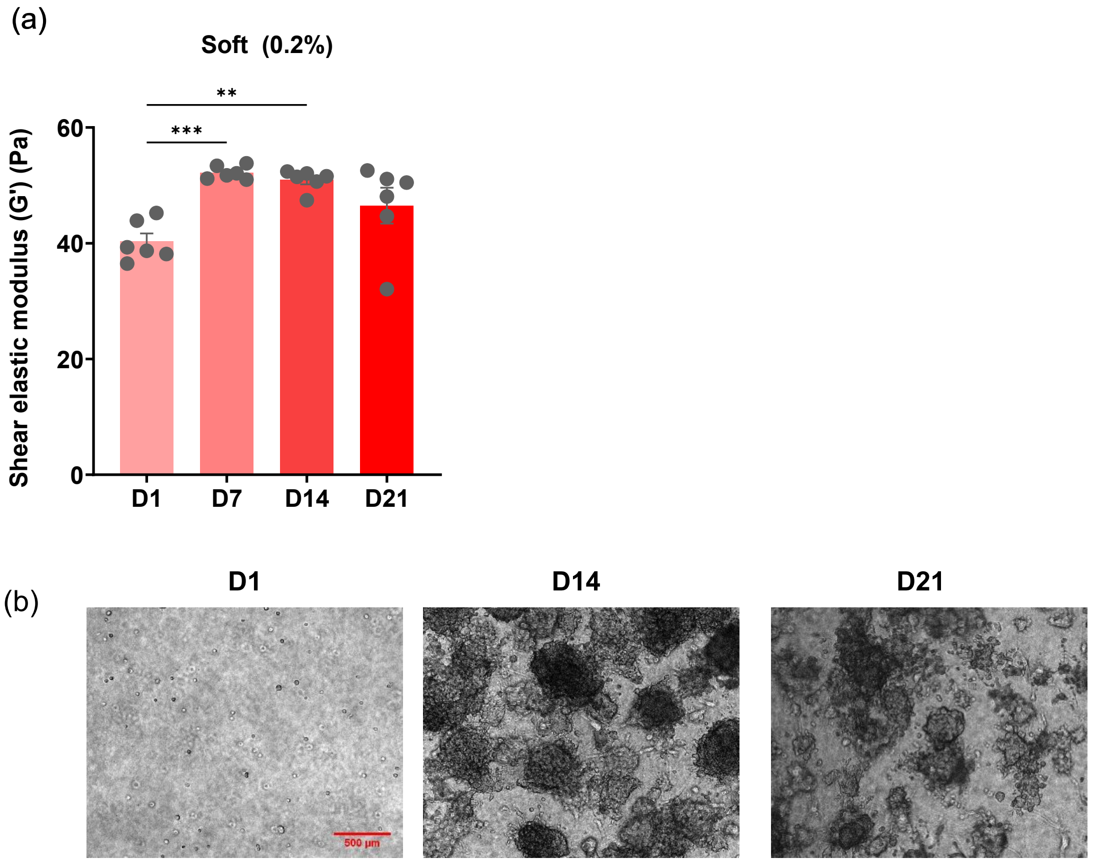

Supp Figure 4. (a) Stiffness measured by shear rheology of soft collagen I hydrogels with ACHN cells over 21 days. One-way ANOVA and Tukey's multiple comparisons test conducted on all cultures, n=6 per condition. p-values ***p<0.0006 **p<0.0019. Error bars represent SEM. **(b)** Light micrograph images of soft ACHN Collagen I cultures over 21 Days, scale bar (500µm).

(c)

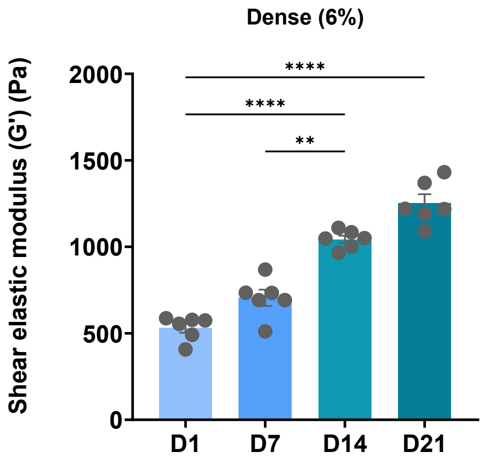

(d)

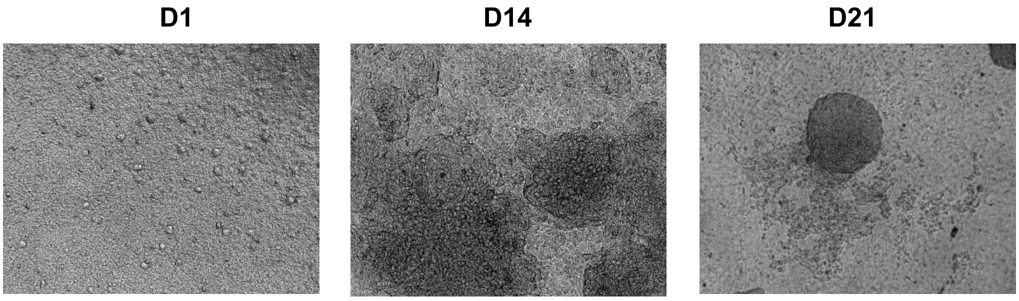

Figure 4. (c) Stiffness measured by shear rheology of dense collagen I gels with ACHN cells over 21 days. p-values ****p<0.0001, **p<0.002. Error bars represent SEM. **(d)** Light micrograph images of dense ACHN Collagen I cultures over 21 Days**,** scale bar (500µm).

Supplementary Figure 5

Figure 5. Gene expression panel of ACHN 3D cultures, soft hydrogels and dense collagen I gels, over 21 days. One-way ANOVA and Tukey's multiple comparisons tests conducted. Error bars represent SEM. p-values ****p<0.0001, ***p<0.0008, **p<0.001, *p<0.02.

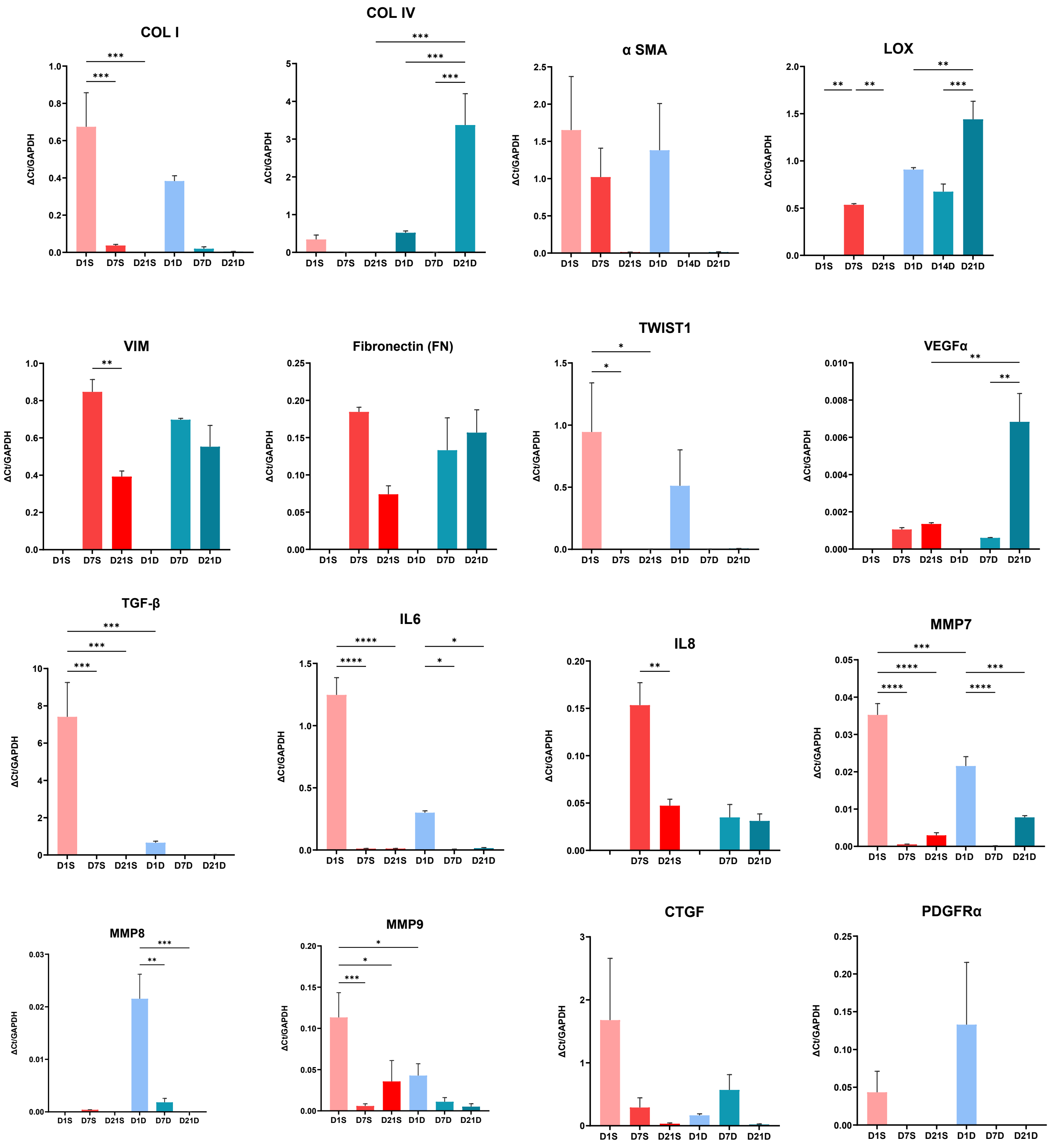

Any markers or values not included in Figure 5 are due to lack of valid expressions being obtained for specific time points of gene markers.

Supplementary Figure 6

786-O (Figure 6)

(a)

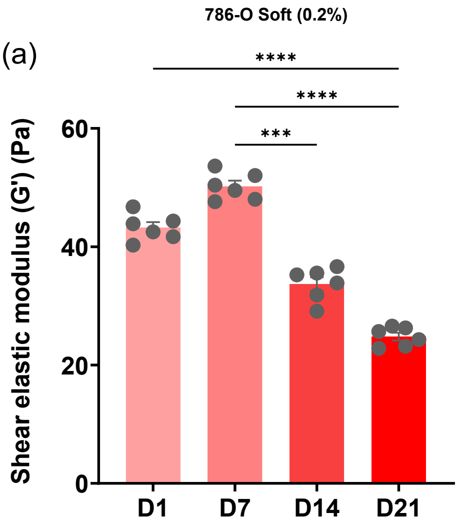

(b)

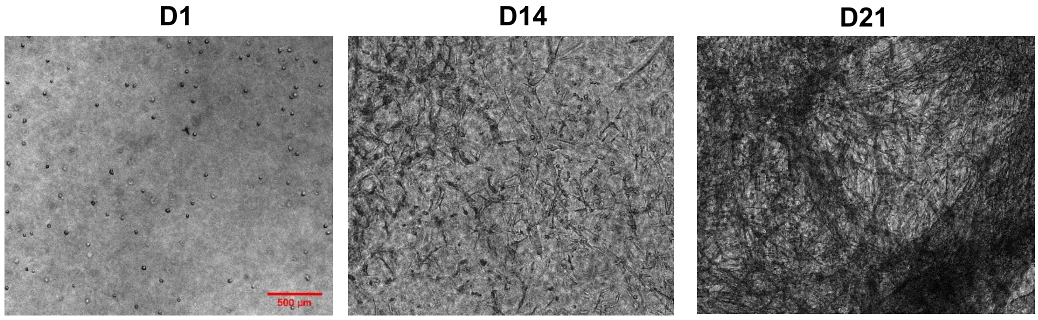

Figure 6. (a) Stiffness measured by shear rheology of soft collagen I hydrogels with 786-O cells over 21 days. p-values ****p<0.0001, ***p<0.0006. Error bars represent SEM. **(b)** Light micrograph images of soft 786-O Collagen I cultures over 21 Days, scale bar (500µm).

(c)

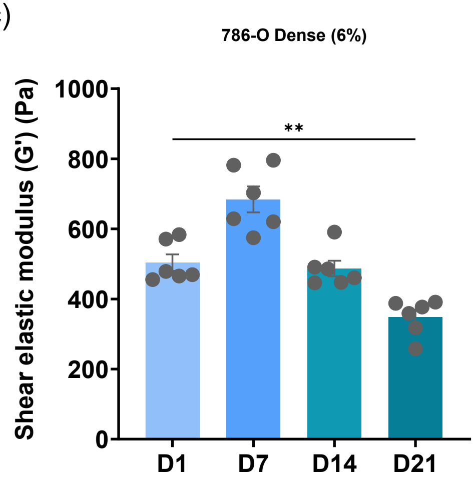

(d)

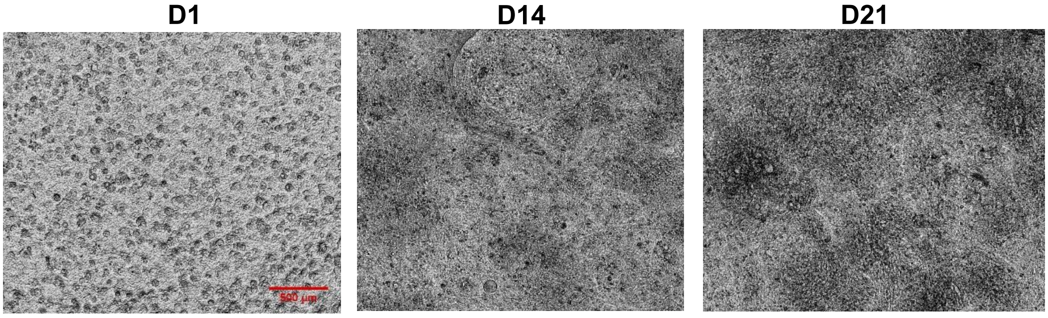

Figure 6. (c) Stiffness measured by shear rheology of soft collagen I hydrogels with 786-O cells over 21 days. p-values $**p<0.004$. Error bars represent SEM. **(d)** Light micrograph images of soft 786-O Collagen I cultures over 21 Days.

Supplementary Figure 7

Figure 7. Gene expression panel of 786-O 3D cultures, soft hydrogels and dense collagen I gels, over 21 days. One-way ANOVA and Tukey's multiple comparisons tests conducted. Error bars represent SEM. p-values ****p<0.0001, ***p<0.0006, **p<0.06, *p<0.02.

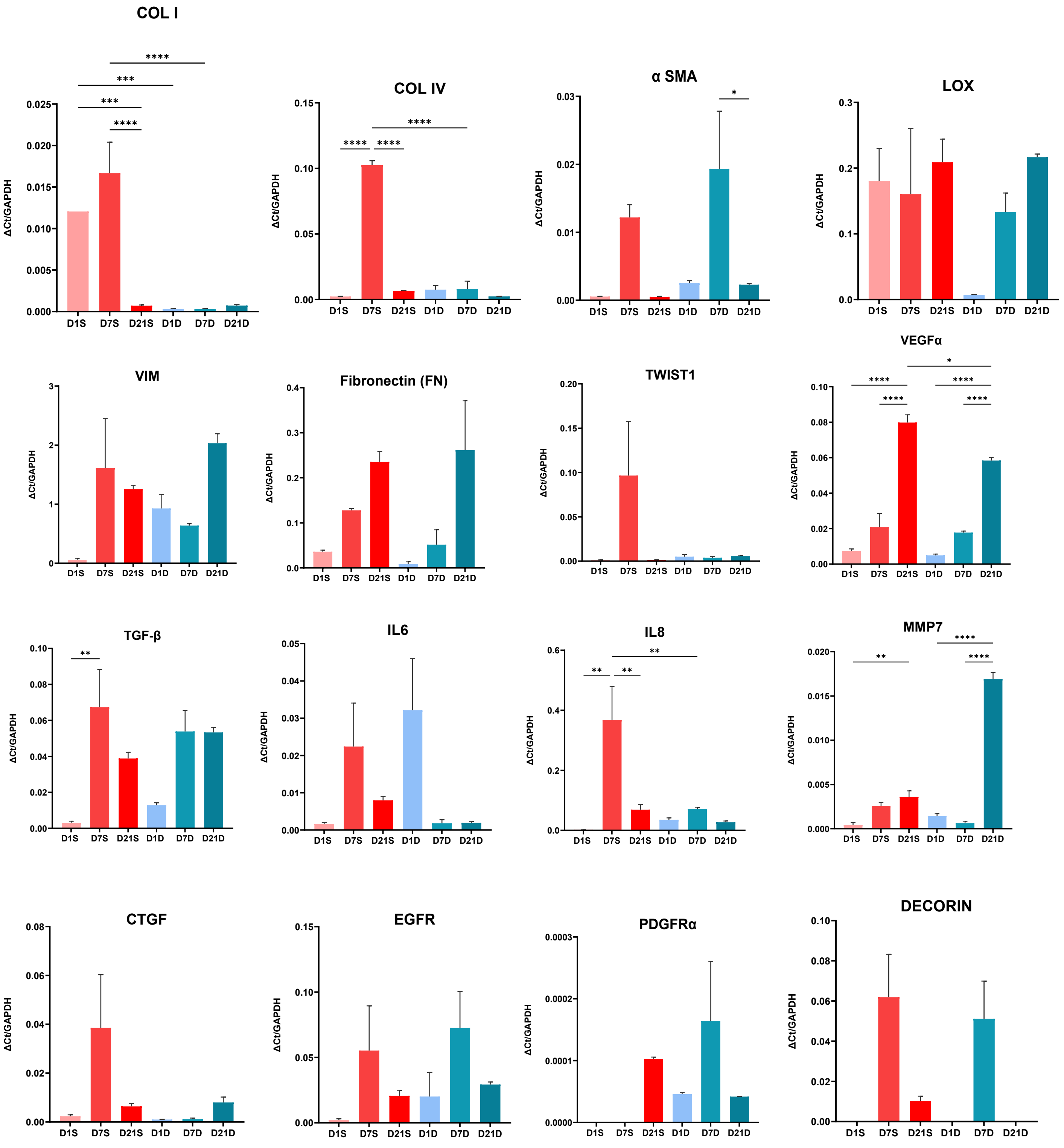

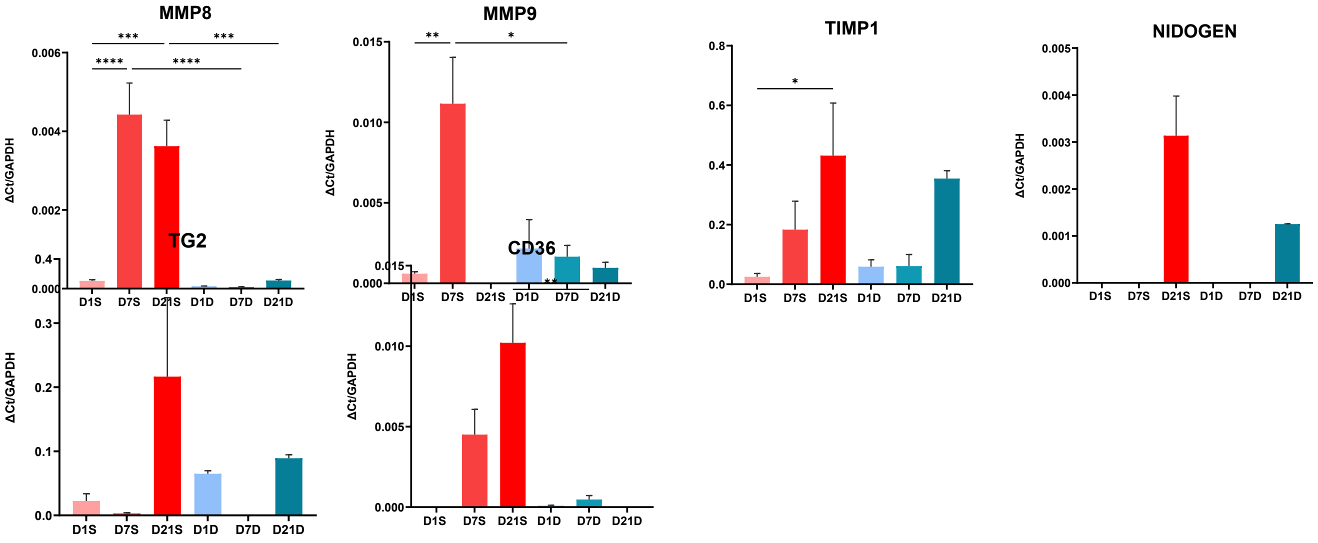

Any markers or values not included in Figure 7 are due to lack of valid expressions being obtained for specific time points of gene markers.
